# Enzymatic Characterization of Recombinant Food Vacuole Plasmepsin 4 from the Rodent Malaria Parasite *Plasmodium berghei*

**DOI:** 10.1371/journal.pone.0141758

**Published:** 2015-10-28

**Authors:** Peng Liu, Arthur H. Robbins, Melissa R. Marzahn, Scott H. McClung, Charles A. Yowell, Stanley M. Stevens, John B. Dame, Ben M. Dunn

**Affiliations:** 1 Department of Biochemistry and Molecular Biology, University of Florida, College of Medicine, Gainesville, Florida, United States of America; 2 Protein Core, Interdisciplinary Center for Biotechnology Research, University of Florida, College of Medicine, Gainesville, Florida, United States of America; 3 Department of Infectious Diseases and Pathology, University of Florida, College of Veterinary Medicine, Gainesville, Florida, United States of America; Russian Academy of Sciences, Institute for Biological Instrumentation, RUSSIAN FEDERATION

## Abstract

The rodent malaria parasite *Plasmodium berghei* is a practical model organism for experimental studies of human malaria. Plasmepsins are a class of aspartic proteinase isoforms that exert multiple pathological effects in malaria parasites. Plasmepsins residing in the food vacuole (FV) of the parasite hydrolyze hemoglobin in red blood cells. In this study, we cloned *PbPM4*, the FV plasmepsin gene of *P*. *berghei* that encoded an N-terminally truncated pro-segment and the mature enzyme from genomic DNA. We over-expressed this *Pb*PM4 zymogen as inclusion bodies (IB) in *Escherichia coli*, and purified the protein following *in vitro* IB refolding. Auto-maturation of the *Pb*PM4 zymogen to mature enzyme was carried out at pH 4.5, 5.0, and 5.5. Interestingly, we found that the *Pb*PM4 zymogen exhibited catalytic activity regardless of the presence of the pro-segment. We determined the optimal catalytic conditions for *Pb*PM4 and studied enzyme kinetics on substrates and inhibitors of aspartic proteinases. Using combinatorial chemistry-based peptide libraries, we studied the active site preferences of *Pb*PM4 at subsites S1, S2, S3, S1’, S2’ and S3’. Based on these results, we designed and synthesized a selective peptidomimetic compound and tested its inhibition of *Pb*PM4, seven FV plasmepsins from human malaria parasites, and human cathepsin D (hcatD). We showed that this compound exhibited a >10-fold selectivity to *Pb*PM4 and human malaria parasite plasmepsin 4 orthologs versus hcatD. Data from this study furthesr our understanding of enzymatic characteristics of the plasmepsin family and provides leads for anti-malarial drug design.

## Introduction

*Plasmodium berghei* is one of the four malaria parasite species that infect rodents [1]. Despite their phylogenetic distance [2], the murine parasites seem to share many biological characteristics with the human species [3–5]. *In vitro* conditions for continuous cultivation of both *P*. *berghei* and *P*. *falciparum*, the most deadly human malaria parasite species, have been well developed [6–9], allowing direct comparison of drug susceptibility of the two species. Indeed, cultured *P*. *berghei* and *P*. *berghei*-infected mice have served as widely used models for anti-malarial drug screening and development of vaccines against malaria [10–20].

Plasmepsins are a class of aspartic proteinases that function in different stages of the life cycle of the malaria parasite *Plasmodium spp*. [21–28]. Genomic analyses of seven human and murine parasites, including *P*. *berghei*, result in identification of seven groups of plasmepsins [29, 30]. One group of plasmepsins function in the food vacuole (FV), a parasite organelle of acidic pH, and are therefore known as FV plasmepsins. The major role of FV plasmepsins involves hydrolyzing hemoglobin, the major cytosolic protein of erythrocytes of vertebrate hosts, to peptides [21]. Plasmepsin-mediated hemoglobin catabolism may provide nutrients [31, 32], maintain osmotic balance [33], and/or make space for the development and growth of the parasites [34]. While four FV plasmepsin genes cluster on the chromosome 14 of *P*. *falciparum*, encoding *Pf*PM1, 2, 4 and a histo-aspartic proteinase, *Pf*HAP, there is only one identified FV plasmepsin thus far in each of the other three human malaria parasites: *Pv*PM4 of *P*. *vivax*, *Po*PM4 of *P*. *ovalae* and *Pm*PM4 of *P*. *malariae* [30].

Comparative genomics analyses indicate that in *P*. *berghei*, one plasmepsin gene, *PbPM4*, shares the highest sequence identity with the FV plasmepsins of human-infecting *Plasmodium spp*. [30]. Located on chromosome 10, *PbPM4* encodes a single polypeptide of 450 amino acids in length comprising an N-terminal 124 amino-acid-long pro-segment and the mature enzyme (S1 Fig) [29, 30]. A growing body of evidence showed that *Pb*PM4 plays a critical role in rodent malaria pathogenesis in that *Pb*PM4-knockout (KO) *P*. *berghei* manifests attenuated virulence and induces protective immunity in the host against wild-type parasites [35–37].

Enzymatic and structural characterization of FV plasmepsins often relied on recombinant expression of truncated zymogen forms lacking a putative trans-membrane motif residing at the N-terminus of the pro-segment, whose presence is typically associated with lower protein yields in *Escherichia coli*, possibly due to its toxicity to the cell [38–40]. In this study, we first cloned, expressed, purified and enzymatically characterized a recombinant, N-terminally truncated zymogen form of *Pb*PM4 lacking the potential trans-membrane motif. In particular, we showed that this semi-pro*Pb*PM4 exhibited catalytic activity even in the presence of a shortened, 48 amino acid-long pro-segment. We identified the optimal catalytic conditions for both zymogen and mature enzyme, and determined kinetic parameters of *Pb*PM4 on varying peptide substrates and inhibitors. Next, we investigated the primary subsite preferences of *Pb*PM4 at S1 and S1’, and the secondary subsite preferences at S3, S2, S2’ and S3’, using two sets of combinatorial peptide libraries. Based on the results here and previous studies [41, 42], we designed a peptidomimetic inhibitor with selectivity to *Pb*PM4 versus the homologous human aspartic proteinase cathepsin D (hcatD). We then synthesized this compound (compound 1), determined its inhibitory effects on *Pb*PM4 and hcatD as well as six FV plasmepsins from four human malaria parasites, and showed that compound 1 had a >10-fold selectivity to *Pb*PM4 and the four plasmepsin 4 homologs of human malaria parasites versus hcatD. Results from this study extend our understanding of active site preferences of the plasmepsin family and offer clues to future anti-malarial drug design.

## Materials and Methods

### Cloning

The sequence encoding the C-terminal 48 amino acid residues of the pro-segment plus the 326 amino acid-long mature enzyme was cloned from *P*. *berghei* ANKA strain genomic DNA. The 1.1 kb DNA fragment was amplified by polymerase chain reaction (PCR) using the primers 5’-CCGGAATTCGGATCCGAATATTTAACAATTCG-3’ (forward), and 5’-CCGGAATTCGGATCCTTAGTTTTTTGCAACTGCAAAAAC-3’ (reverse). The purified PCR product was inserted into the *Bam*HI cloning site of the pET-3a expression vector (69418; EMD Millipore, Billerica, MA), and its sequence was verified by DNA sequencing analysis (Interdisciplinary Center for Biotechnology Research, University of Florida, Gainesville, Florida). The construct was transformed into BL21 Star (DE3) pLysS *E*. *coli* expression cell line (C6020-03; Invitrogen, Carlsbad, CA).

### Expression and inclusion body preparation

BL21 Star (DE3) pLysS *E*. *coli* cells harboring the semi-pro*Pb*PM4-pET-3a construct were inoculated into Luria Broth media containing 34 μg/mL chloramphenicol and 50 μg/mL ampicillin. Cells were grown at 37°C with a shaking speed of 250 rpm until A_600_ reached 0.6. Isopropyl β-D-1-thiogalactopyranoside (IPTG) at the final concentration of 1 mM was introduced to cell culture to induce protein expression.

Cell culture was harvested after 3 hr by centrifugation at 4°C, 13,000 *g*, for 15 min. *E*. *coli* cells were resuspended in ice-cold buffer A (10 mM Tris-HCl, pH8.0; 20 mM magnesium chloride; 5 mM calcium chloride), and lysed by French pressure cell press under 12,000 psi. Inclusion bodies obtained from cell lysate were further purified using the methods previously described for the purification of other plasmepsins [43, 44]. Briefly, a final concentration of 80 Kunitz units/mL of DNase I (M0303S; New England BioLabs, Ipswich, MA) was added to the lysate and incubated at room temperature for 15 min. Five to 10 mL of cell lysate was layered over 10 mL of 27% (w/v) sucrose and centrifuged at 12,000 *g*, 4°C, for 45 min. The pellet was resuspended in buffer 2 (10 mM Tris-HCl, pH 8.0; 1 mM ethylenediaminetetraacetic acid (EDTA); 2 mM β-mercaptoethanol; 100 mM sodium chloride), and 5–10 mL of the resuspension was layered over 10 mL of 27% (w/v) sucrose, and centrifuged at 12,000 *g*, 4°C, for 45 min. The pellet was then resuspended in buffer 3 (50 mM Tris-HCl, pH 8.0; 5 mM EDTA; 2.5 mM β-mercaptoethanol; 0.5% (v/v) Triton-X-100), and centrifuged at 12,000 *g*, 4°C, for 15 min. The resulting pellets were washed with buffer 4 (50 mM Tris-HCl, pH 8.0; 5 mM EDTA; 2.5 mM β-mercaptoethanol), and harvested by centrifugation at 12,000 *g*, 4°C, for 15 min. The purified inclusion bodies were resuspended in buffer 5 (10 mM Tris-HCl, pH 8.0; 1 mM EDTA) to a final concentration of 100 mg/mL, and stored at -80°C.

### Refolding and purification

*In vitro* protein refolding and subsequent purification were performed following the experimental procedures previously described [42]. Briefly, inclusion bodies, after thawing on ice, were resuspended and added dropwise to a freshly prepared denaturation buffer (deionized 6 M urea; 50 mM sodium phosphate, pH 8.5; 500 mM sodium chloride). Protein was denatured at room temperature for 2 hr with a Teflon-coated bar stirring at 90 rpm. Any undissolved material was removed by centrifugation at 13,000 *g*, 4°C for 30 min, and the supernatant was filtered through a 0.22 μm filter. The filtered supernatant was dialyzed against 20 mM Tris-HCl, pH 8.0 at 4°C. The dialysis buffer was changed every 6 hr three more times. The resulting dialysate was centrifuged at 13,000 *g*, 4°C for 30 min, and filtered through a 0.22 μm membrane to remove any precipitates.

The semi-pro*Pb*PM4 was initially purified from the soluble dialysate using a HiTrap Q HP 5 mL anion exchange column (17-1154-01; GE healthcare, Pittsburgh, PA). Briefly, the column was first equilibrated with elution buffer A (20 mM Tris-HCl, pH 8.0), then elution buffer B (20 mM Tris-HCl, pH 8.0; 500 mM sodium chloride), and then buffer A. The dialysate was then loaded onto the column, washed with elution buffer A, and the protein was subsequently eluted with a gradient of 0–500 mM sodium chloride. The protein concentration and catalytic activity of each fraction were tested using a Cary 50 Bio UV-Visible spectrophotometer (Agilent Technologies, Foster City, CA). Protein concentration was measured using OD_280_. The catalytic activity assay was carried out at 37°C by pre-incubating protein in 100 mM sodium citrate, pH 5.0 for 5 min, then mixing with 40 μM of a chromogenic peptide substrate: Lys-Pro-Ile-Leu-Phe*Nph-Arg-Leu (Nph = *para*-nitrophenylalanine and * represents the bond where cleavage occurs), and immediately measuring the initial cleavage velocity. The OD_280_ and catalytic activity peaks overlapped at the elution peak corresponding to a sodium chloride concentration of 300 mM.

The final step of protein purification was carried out using size exclusion chromatography. The peak fractions from anion exchange chromatography were pooled and concentrated using Vivaspin 15R centrifugal concentrators (MWCO = 5 kDa, VIVASCIENCE, Littleton, MA) until OD_280_ reached 1.5. The concentrated samples were centrifuged at 24,000 *g*, 4°C for 10 min to remove any precipitates. Three mL of the concentrated sample were injected into a HiLoad 16/60 Superdex 75 column (17-1068-01, GE healthcare). The protein concentration and catalytic activity of each fraction were tested as described above. Fractions comprised of the catalytic activity peak were pooled and stored at 4°C.

### Auto-maturation and catalysis optimization

For auto-maturation, a purified recombinant semi-pro*Pb*PM4 sample was evenly allocated into six aliquots, and each aliquot was incubated at 37°C with one of the following 500 mM acidic buffers of one fifth its volume: sodium formate, pH 3.5; sodium formate, pH 4.0; sodium acetate, pH 4.5; sodium citrate, pH 5.0; sodium citrate, pH 5.5; and sodium phosphate, pH 6.0. For all the six conditions, an equal volume of sample was withdrawn at each of the following designated incubation time periods: 0, 5, 10, 30, 60, 120, 240, 480 min and overnight. Into each of the withdrawn samples 5× Laemmli sample buffer (500 mM Tris-HCl, pH 8.0; 8% (w/v) sodium dodecyl sulfate (SDS); 0.01% (w/v) Coomassie brilliant blue, 0.1% (v/v) phenol red; 25% (v/v) glycerol; 5% (v/v) β-mercaptoethanol) was immediately added, and the resulting sample was then boiled for 10 min. The auto-maturation of zymogen to mature enzyme was analyzed by SDS-polyacrylamide gel electrophoresis (PAGE).

For determining the optimal catalytic conditions, purified semi-pro*Pb*PM4 was treated similarly as described above. Each withdrawn sample, in this case, was immediately mixed with 100 μM of the peptide substrate Lys-Pro-Ile-Leu-Phe*Nph-Arg-Leu. The initial cleavage velocities were measured on a Cary 50 Bio UV-Visible spectrophotometer, and were normalized to the highest enzymatic cleavage velocity of all the tested reactions, which was set to 100 percent. Assays at each time period were performed three times, from which the mean and standard error of the mean (SEM) were determined. The combined pH and incubation time that allowed the enzyme to show the highest catalytic activity was defined as the optimal catalytic conditions.

### N-terminal sequencing analysis

Protein samples were electrophoresed on a 10% Tris-Tricine polyacrylamide gel, and were then transferred onto a polyvinylidene difluoride membrane using transfer buffer: 10 mM MES, pH 6.0, plus 20% methanol, under the following settings: 90 volts (constant), room temperature, 2 hr. The N-terminal amino acid sequencing analyses were performed based on the Edman degradation method [45] using an Applied Biosystems 470A protein sequencer at the Protein Chemistry Core Facility, University of Florida, Gainesville, Florida.

### Kinetic analysis

#### Substrate, inhibitor and enzyme preparation

Chromogenic peptide substrates were dissolved in a solution containing 20% dimethyl sulfoxide (DMSO), 10% formic acid, and 70% distilled deionized water to generate stock solutions. Inhibitor stock solutions were prepared in 100% DMSO. The concentrations of the stock solutions were determined by amino acid analysis [46].

Kinetic assays were set up in 500 mM sodium citrate, pH 5.0 at 37°C with a 5-min pre-equilibrium time with buffer. To study kinetics of the zymogen form, assays were set up in 500 mM sodium citrate, pH 5.5 at 37°C with a 5-min pre-equilibrium time. All enzymatic reactions were carried out at 37°C.

#### Substrate hydrolysis and *K*_m_

The substrate hydrolysis was analyzed by spectroscopy and defined as the decrease of the average absorbance between 284–324 nm [47, 48]. Pre-equilibrated enzyme was mixed with substrate at least six different concentrations (μM). The initial cleavage velocities (AU/sec) of these reactions were immediately measured on a Cary 50 Bio UV-Visible spectrophotometer. The initial cleavage rates (M/sec) were converted from the observed velocities (AU/sec) by dividing the observed velocities by the total absorbance changes upon complete enzymatic digestion of the substrate of known concentrations (i.e., AU/M). The *V*_max_ and *K*_m_ were determined from the converted initial rates (*v*) and corresponding substrate concentrations ([*S*]) by the equation:

v=Vmax*[S]/(Km+[S]) (1)

and Marquardt analysis [49] under the single substrate option of the enzyme kinetic module 1.0 of SigmaPlot 2000 (Version 6.10) (Systat Software Inc., San Jose, CA).

#### Active site titration and *k*_cat_

The total concentration of active enzyme, [*E*]_tot_, was determined by titrating *Pb*PM4 enzyme with the tight binding, competitive, aspartic proteinase inhibitor pepstatin A. A constant amount of pre-equilibrated enzyme was mixed either with 100 μM of the peptide substrate Lys-Pro-Ile-Leu-Phe*Nph-Arg-Leu or with both 100 μM of substrate and pepstatin A at a series of different concentrations. The initial cleavage rates of the substrate at different inhibitor concentrations were immediately measured afterwards using a Cary 50 Bio UV-Visible spectrophotometer. [*E*]_tot_ was determined by fitting the initial cleavage velocities (*v*) and pepstatin A concentrations ([*I*]) into the Henderson equation [50, 51] under the tight-binding inhibition option of the enzyme kinetic module 1.0 of SigmaPlot 2000. *k*_cat_ (sec^-1^) was calculated from the equation:

kcat=Vmax/[E]tot (2)

#### Dissociation constants (*K*_i_)

Dissociation constants were determined as previously described [42]. Briefly, for tight-binding (*K*_i_ = 50 pM– 10 nM) competitive inhibition, *K*_i_ was determined by fitting the initial cleavage velocities (*v*) and inhibitor concentrations ([*I*]) into the following equation [52] using the Enzfitter1.05 program (BioSoft, Cambridge, UK):

$$\begin{array}{l} v=\left\{\left(0.5*V_{\text{max}}/\left[E\right]\right)/\left(K_{m}/\left[E\right]+1\right)\right\}*\\ \left\{\left(\left[E\right]-\left[I\right]-K_{iap}\right)+\sqrt{\left(\left[E\right]-\left[I\right]-K_{iap}\right)*\left(\left[E\right]-\left[I\right]-K_{iap}\right)+\left(4*\left[E\right]*K_{iap}\right)}\right\} \end{array}$$ (3)

where

$$K_{iap}=K_{i}*\left(\left[S\right]/K_{m}+1\right)$$ (4)

and the enzyme concentration ([*E*]), substrate concentration ([*S*]), *K*_m_ and *V*_max_ were constant and known. For non-tight-binding (*K*_i_ = 50 nM– 10 μM) competitive inhibition, *K*_i_ was determined by fitting the initial cleavage velocities (*v*), substrate ([*S*]) and inhibitor ([*I*]) concentrations into the equation:

$$v=V_{\text{max}}*\left[S\right]/\left\{\left[S\right]+K_{m}*\left(1+\left[I\right]/K_{i}\right)\right\}$$ (5)

under the single substrate—single inhibitor (competitive) option of the enzyme kinetic module 1.0 of SigmaPlot 2000.

### Subsite preferences

#### Combinatorial peptide library

Two sets of combinatorial chemistry-based peptide libraries, which were designed based on previous substrate specificity studies of aspartic proteinases [44, 53, 54], were used to investigate the S3 –S3’ subsite preferences of *Pb*PM4. The P1 combinatorial library is comprised of octa-peptides of the sequence Lys-Pro-Xaa-Glu-P1*Nph-Xaa-Leu (Nph = *para*-nitrophenylanaline, and * represents the peptide bond where aspartic proteinase digestion occurs). This library contains 19 peptide pools, each of which is named after the amino acid residue at the P1 position. The 19 residues at P1 include 18 natural amino acids, omitting methionine and cysteine, and norleucine. Within each pool, a mixture of these 19 amino acids (Xaa) is incorporated in both the P3 and P2’ positions, which results in a total of 361 peptide species (19 × 19) for an individual pool, and 6859 for the whole library (19 × 19 ×19). The P1’ combinatorial library is similarly designed except that peptides have the sequence Lys-Pro-Ile-Xaa-Nph*P1’-Gln-Xaa, and therefore each pool is named after the residue at P1’ and the mixture of those 19 amino acids (Xaa) is accommodated at P2 and P3’. The P1 and P1’ library synthesis has been described in detail previously [41].

#### Primary subsite preferences—spectroscopic assays

The primary subsite preferences of *Pb*PM4 at the S1 and S1’ positions were determined by analyzing the initial cleavage velocities of peptide pools. Detailed experimental procedures were described previously [42]. Briefly, each of the 19 lyophilized peptide pools was dissolved in filtered distilled deionized water to make 1.25 mM stock solutions, which were then filtered through 0.45 μm Costar cellulose acetate tube filters by centrifugation at 20,000 *g* at room temperature for 5 min to remove any undissolved material. Meanwhile, 1 μM of the semi-pro*Pb*PM4 was incubated in 100 mM sodium citrate, pH 5.0, at 37°C for 5 min to convert to mature enzyme. This enzyme preparation was then mixed with peptide substrates. The initial cleavage velocities on 100 μM of peptide pools were measured at 37°C using a Cary 50 Bio UV-Visible spectrophotometer, and were then normalized to the highest velocity among the 19 pools, which was set to 100 percent. Experiments were performed three times, from which the mean values and SEM were determined.

#### Secondary subsite preferences—liquid chromatography/mass spectrometry

The secondary subsite preferences of *Pb*PM4 at the S3, S2, S2’ and S3’ sites were determined by measuring the relative abundance of penta- and tri-peptides produced from proteinase digestion. The three peptide pools from each library that showed the highest cleavage velocities were chosen to study secondary subsite preferences at S3, S2, S2’ and S3’. The complete digestion process of 100 μM of each selected P1 or P1’ library pool was monitored on a Cary 50 Bio UV-Visible spectrophotometer, from which the total alterations in the average absorbance between 284–324 nm were calculated. The time for enzymatic digestion allowing only 5–10% of substrate hydrolysis, i.e., the linear phase of a kinetic reaction, was thus determined. These times were used to perform hydrolysis of the selected peptide pools. The enzymatic reactions were quenched by addition of 1% (v/v) of 14 M ammonium hydroxide to raise pH greater than 8.0, and the digested peptides were subject to liquid chromatography/mass spectrometry (LC/MS) analysis.

The approaches for in-line LC/MS isolation, identification and quantification of peptide products were previously described in detail [42]. Briefly, individual peptides were isolated via capillary reverse phase high performance liquid chromatography using a self-packed 20 cm × 75 μm i.d. Alltima C18 reverse phase column (particle size: 5μm) (Alltech Associates, Deerfield, IL) in combination with an Ultimate Capillary HPLC system (LC Packings, San Francisco, CA) operated at a flow rate of 200 nL/min. In-line MS analyses of the column eluate were performed using a Thermo-Finnigan LCQ Deca quadrupole ion trap mass spectrometer (Thermo Electron Corp, San Jose, CA) under the electrospray ionization mode (ESI) with the following technical parameters: sheath gas (N2) = 0, aux gas (N2) = 0, spray voltage = 2 kV, capillary temperature = 175°C, capillary voltage = 33 V and tube lens offset = 20 V. Peptide quantity was determined by integrating the area under the curve of [M+H]^+^ and [M+2H]^2+^ ions for the penta-peptide cleavage products, and that of [M+H]^+^ ions for the tri-peptide products via the Qual Browser program of the X-Calibur 1.3 software package (Applied Biosystems, Foster City, CA). For each peptide pool, the LC/MS analysis was repeated three to four times, from which the average relative abundances and SEM were calculated. The favored residues at P3, P2, P2’ and P3’ are those whose residing penta- or tri-peptides are the most abundant.

### Peptidomimetic inhibitor design and inhibition analyses

#### Inhibitor design

For the design of the combinatorial chemistry inhibitor of *Pb*PM4, we selected the P1 and P1’ amino acid substitutes based on two factors and one observation: factor 1 –the initial velocity of *Pb*PM4-catalyzed cleavage of each octa-peptide pool, factor 2 –the difference between the initial velocity of *Pb*PM4-catalyzed cleavage of a peptide pool and the velocity of hcatD-catalyzed cleavage of that pool, and an observation that auto-maturation of *Pb*PM4 zymogen occurs in the pro-segment where bulky hydrophobic residues are accommodated at the S1 subsite, and hydrophobic or positively charged, but not negatively charged residues are accommodated at the S1’ subsite.

To select the P3 and P2’ amino acid substitutes, we analyzed the cleavage products from *Pb*PM4- and hcatD-catalyzed digestion of the P1-phenylalanine pool, the most favored by hcatD (Tables A and G in S1 File), based on two factors: 1) the relative abundance of each peptide product from *Pb*PM4-catalyzed cleavage of the pool, and 2) the difference between the relative abundance of each peptide product from *Pb*PM4-catalyzed cleavage of the pool and that from hcatD-catalyzed cleavage. The same approach was used to select the P2 and P3’ amino acid substitutes except that the cleavage products from *Pb*PM4- and hcatD-catalyzed digestion of the P1’-phenylalanine pool, the most favored by hcatD (Tables B and H in S1 File), were analyzed.

To help better understand the rationale for compound design, the original data showing primary and secondary subsite preferences of *Pb*PM4 and hcatD (Tables A-F in S1 File) were reported.

A compound (compound 1) comprised of such selected residues was synthesized with the scissile peptide bond between P1 and P1’ modified as a non-cleavable methyleneamino [-CH_2_-NH-].

#### Recombinant plasmepsins and hcatD preparation

Besides *Pb*PM4, the cloning, expression and purification of recombinant human FV plasmepsins, including *Pf*PM1, 2 and 4, *Po*PM4, *Pv*PM4 and *Pm*PM4, and hcatD were performed according to experimental procedures described in previous reports [42–44, 55–57]. Briefly, genes encoding N-terminally truncated semi-pro-enzymes were cloned from genomic DNA, or intra-erythrocyte stage cDNA library of *Plasmodium spp*. (e.g., in the case of *Pf*PM1), and inserted to pET expression vectors (EMD Millipore). Protein expression, inclusion body preparation and *in vitro* protein refolding were performed as described above for *Pb*PM4. Proteins were purified using anion exchange chromatography. Purified enzymes were subject to active site titration using pepstatin A, and were found to be 100% active.

#### Inhibition analyses

The enzymes were prepared for kinetic assays: purified pro*Pb*PM4 was pre-incubated in 100 mM sodium citrate, pH 5.0, at 37°C for 5 min; purified mature *Pf*PM1 was pre-incubated in 100 mM sodium citrate, pH 5.5, at 37°C for 3 min; all the other plasmepsins were pre-incubated in 100 mM sodium formate, pH 4.5, at 37°C for 5 min to convert zymogen to mature enzyme before the addition of substrates [43, 44, 47]. Pre-incubation of hcatD was carried out in 200 mM sodium formate, pH 3.7, at 37°C for 5 min. Compound 1 was dissolved in 100% DMSO, and filtered through a 0.45 μm Costar cellulose acetate tube filter by centrifugation at 20,000 *g*, room temperature for 5 min to remove any particulate. The concentration of compound 1 was determined by amino acid analysis. Dissociation constants (*K*i) were determined as described above.

## Results

### Expression, *in vitro* refolding, and purification

**Fig 1:**
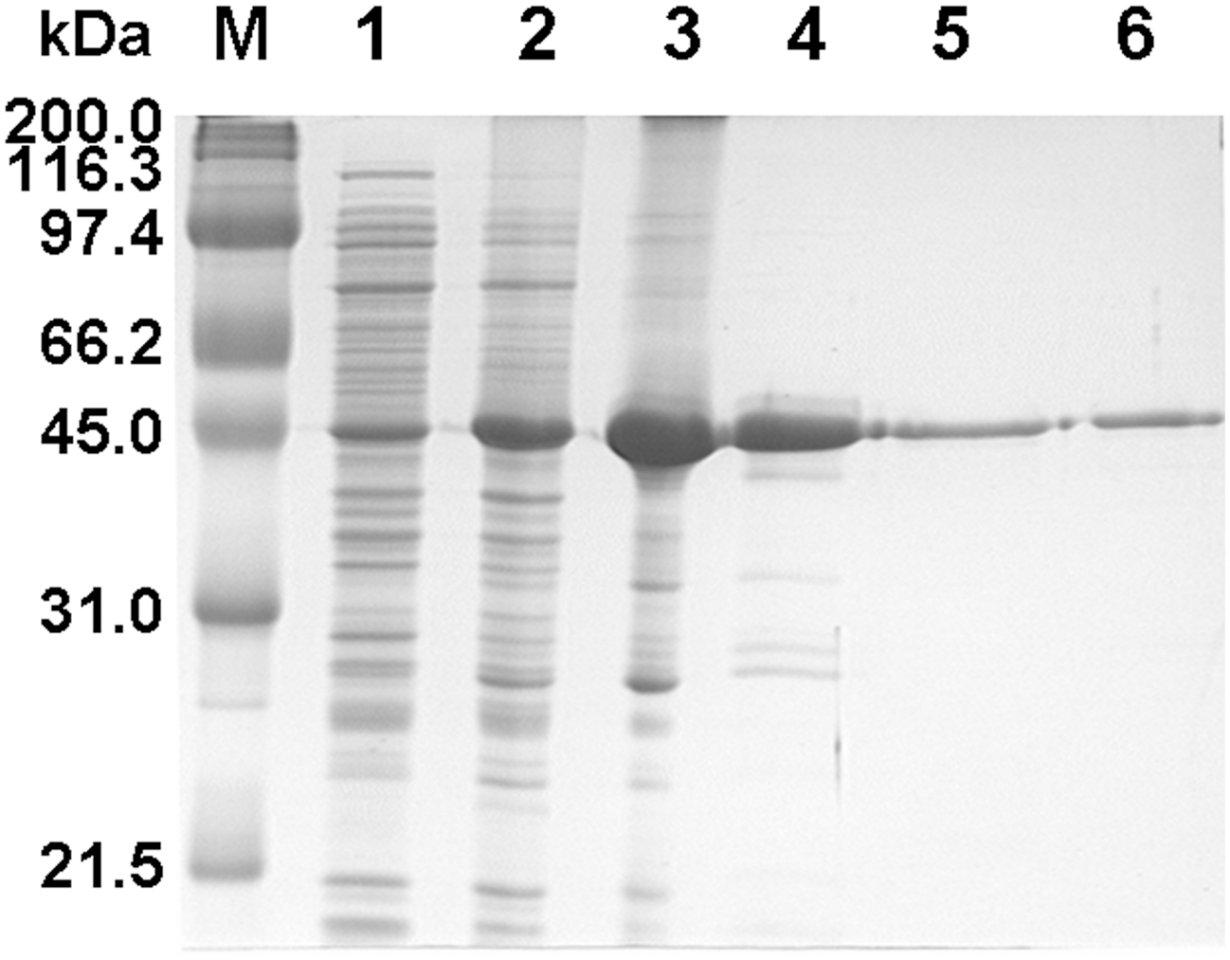
SDS-PAGE analysis of over-expression and purification of recombinant semi-pro*Pb*PM4. M: molecular weight markers; 1: lysates of pre-IPTG-induced *E*. *coli* in 20 μL of cell suspension (OD_600_ = 0.61); 2: lysate of post-IPTG-induced *E*. *coli* in 8.2 μL of cell suspension (OD_600_ = 1.48); 3: purified, pro*Pb*PM4-enriched inclusion body (protein loading in lane: ~30 μg); 4: soluble dialysate following filtration of the *in vitro* refolding products (protein loading in lane: 20 μg); 5: anion exchange chromatography-purified pro*Pb*PM4 (protein loading in lane: 5 μg); 6: size exclusion chromatography-purified pro*Pb*PM4 (protein loading in lane: 5 μg). kDa: kilo-Daltons.

**Fig 2:**
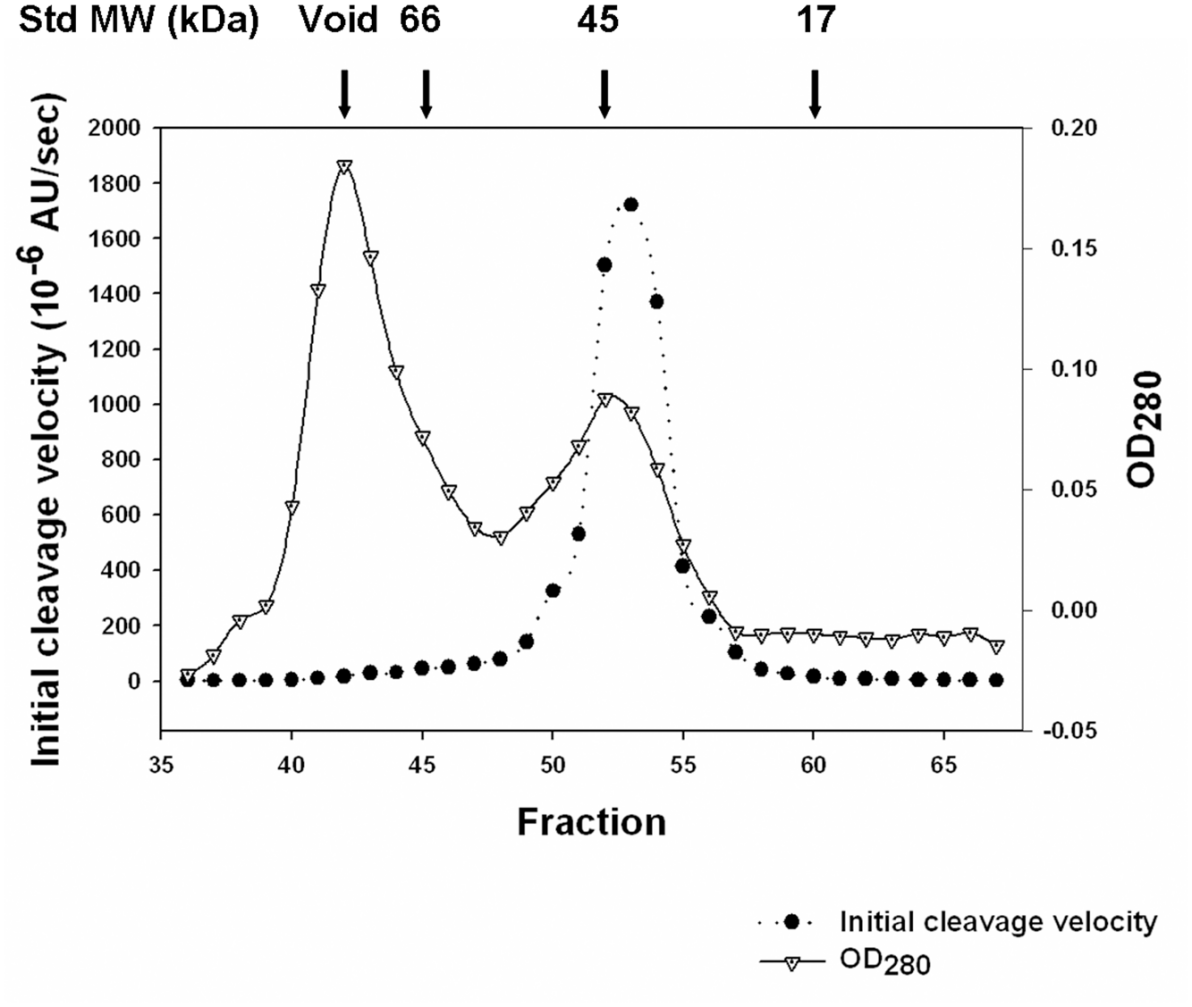
Size exclusion purification chromatogram of the recombinant protein. Catalytic activity and concentration of total protein contained in fractions 36–67 were measured and plotted against the fraction numbers. The early eluted protein, whose concentration was peaked at fraction 42, showed no apparent catalytic activity; in contrast, the later eluted protein had the concentration and catalytic activity peaks overlapped at fractions 52–53, which was similar to the elution pattern of a globular protein of 44 kDa. Protein in fraction 53 was subject to SDS-PAGE analysis and was revealed as a single band migrating at ~43 kDa (Fig 1, lane 6), indicating that the peak comprised of fractions 51–55 contains monomeric semi-pro*Pb*PM4. The elution patterns of globular proteins serving as standard were marked above the chromatogram. Concentrations and catalytic activities of the fractions 1–35 and >67 were also tested, but no peaks were detected. AU = arbitrary unit. Void = void volume.

The semi-pro*Pb*PM4 was recombinantly expressed in *E*. *coli*, and isolated as inclusion bodies. These insoluble materials were denatured, refolded *in vitro*, and purified. Representative SDS-PAGE analysis revealed *Pb*PM4 zymogen at each step of expression and purification (Fig 1). The average yield of inclusion bodies was approximately 15% of the total cell mass, indicative of over-expression (Table 1). Approximately 1.7% (w/w) of the recombinant protein initially exposed to denaturant was ultimately purified by size exclusion chromatography as monomeric pro-enzyme bearing catalytic activity (Fig 2; Table 1).

**Table 1:** Yields of expression and purification of the recombinant semi-pro*Pb*PM4 from one liter culture.

Production and purification steps	Average yields (mg)^a^
Cell pellet (wet)	2780 ± 260^b^
Inclusion body (wet)	420 ± 40^c^
6 M urea denaturation	60 ± 11^d^
Refolded dialysate	13 ± 2.3^d^
Anion exchange chromatography	8.3 ± 1.2^d^
Gel filtration chromatography	1.0 ± 0.2^d^

^a^The product yield of each step was the result from three individual expressions, presented as mean ± SEM.

^b^Weights were directly measured using a balance after centrifugation.

^c^Weights were directly measured using a balance after purification.

^d^The concentration of soluble protein was determined using OD_280_ (ε_280_ = 41,510 M^-1^ cm^-1^, a theoretical value calculated from the sequence of semi-pro*Pb*PM4 using ProtParam [58]).

### Enzymatic characterization

#### Auto-maturation

**Fig 3:**
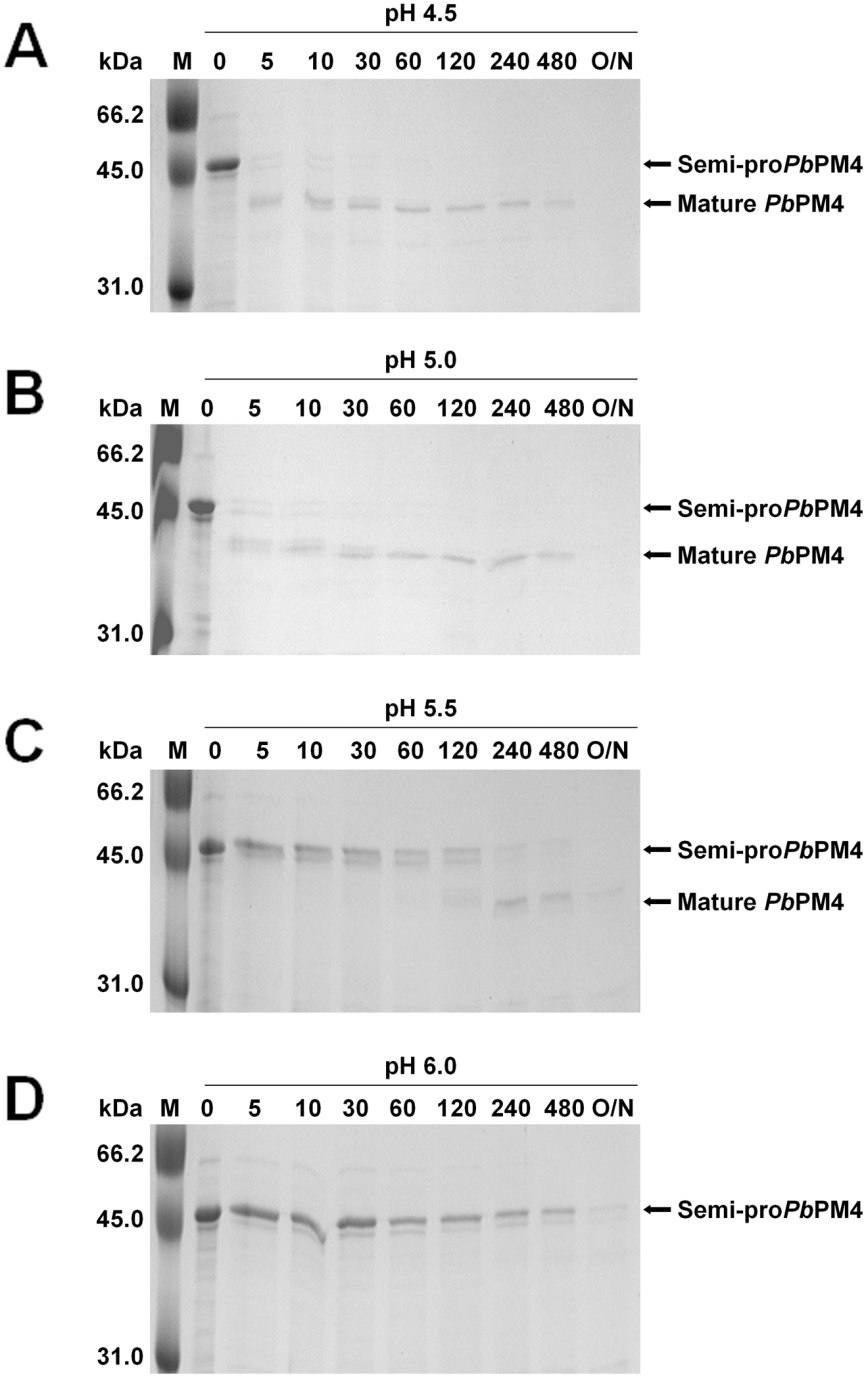
SDS-PAGE analysis of time-resolved auto-maturation of pro*Pb*PM4. All of the auto-maturation assays were performed at 37°C by incubating 2.4 μg of pro*Pb*PM4 in buffer of pH 4.5 (A), pH 5.0 (B), pH 5.5 (C), and pH 6.0 (D). The molecular conversion from zymogen (~43 kDa) to mature enzyme (~36 kDa) was monitored at the time indicated above the gel images. M: molecular weight marker, unit: min, O/N = overnight.

Known FV plasmepsins are capable of conducting auto-maturation *in vitro* to convert zymogens to mature enzymes [59–62]. Here, auto-maturation of the semi-pro*Pb*PM4 was studied at acidic pH 4.5–6.0. Auto-maturation was fully conducted at pH 4.5 and pH 5.0 with an incubation time of 5 min (Fig 3A and 3B). Auto-maturation of the pro-segment at pH 5.5, however, was remarkably delayed such that mature *Pb*PM4 can only be appreciably detected after 2 hr incubation (Fig 3C); whereas enzyme maturation at pH 6.0 was completely halted for at least 12 hr (Fig 3D). In addition, incubation of semi-pro*Pb*PM4 in buffers of pH 3.5 and pH 4.0 resulted in a quick, non-specific degradation process within minutes. These observations suggest that auto-maturation of the recombinant semi-pro*Pb*PM4 is a pH-sensitive, time-dependent process. N-terminal protein sequencing analyses revealed that the cleavage site of the final products converted at pH 4.5, 5.0 and 5.5 was exclusively between Leu117p and Leu118p, implying an enzyme-mediated activation of semi-pro*Pb*PM4.

#### Catalysis optimization

**Fig 4:**
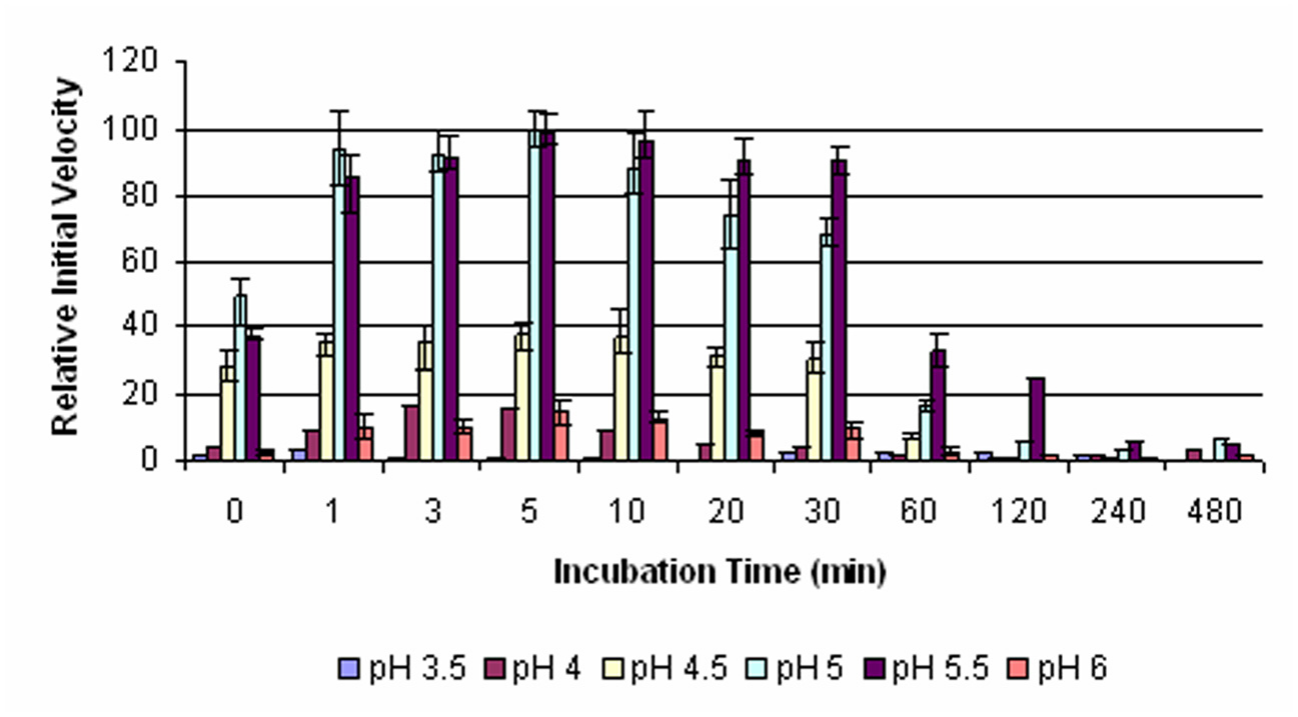
Determination of the optimal conditions for the catalysis of *Pb*PM4. Experiments were performed at 37°C, in buffers of pH 3.5–6.0. The pro*Pb*PM4 was pre-incubated in buffers for the time indicated. Subsequently, enzyme-catalyzed initial hydrolysis rates of a chromogenic substrate were measured and normalized to the maximal initial velocity, which was set to 100 percent.

The catalytic activities of *Pb*PM4 at pH 3.5–6.0 were assessed in a time course assay. The conditions for the enzyme to show highest catalytic activity were determined to be at pH 5.0 and pH 5.5 with a 5 min pre-incubation (Fig 4). In addition, a majority (75–90%) of the maximal catalytic activity was maintained within 30 min pre-incubation under such pH conditions. Interestingly, while semi-pro*Pb*PM4 was largely unprocessed within 2 hr pre-incubation at pH 5.5, exposure at this pH allowed zymogen to gain enzymatic activity despite the presence of the pro-segment (Figs 3C and 4). Overall, the optimal catalytic condition is 5 min pre-incubation at 37°C in 100 mM sodium citrate, pH 5.0 for mature *Pb*PM4, and pH 5.5 for *Pb*PM4 zymogen.

#### Kinetic analysis

We first determined the Michaelis constant (*K*_m_), catalytic constant (*k*_cat_) and catalytic efficiency (*k*_cat_/*K*_m_) of *Pb*PM4 using two chromogenic peptide substrates, which have been used previously to study catalysis of pepsin-like aspartic proteinases [54]. Compared to other FV plasmepsin orthologs of human malaria parasites [44, 56], mature *Pb*PM4 showed similar kinetic profiles on digestion of such substrates (Table 2).

**Table 2:** Kinetic analyses of the cleavage of *Pb*PM4 on chromogenic peptide substrates.

Substrate^a^	*k*_cat_ (s^-1^)	*K*_m_ (μM)	*k*_cat_/*K*_m_ (μM^-1^ s^-1^)
K-P-I-L-F*Nph-R-L^§^	34.9 ± 5.7	2.1 ± 0.2	16.5 ± 2.9
K-P-I-Q-F*Nph-R-L^§^	24.5 ± 1.8	3.8 ± 0.3	6.4 ± 0.7

^a^Nph = *para*-nitrophenylalanine;

* represents the scissile bond where cleavage occurs.

Unlike other FV plasmepsin orthologs of human malaria parasites, *Pb*PM4 showed enzymatic activity in the presence of the semi-pro-segment. To understand whether semi-pro*Pb*PM4 shared similar kinetics of peptide cleavage with the mature enzyme, we monitored zymogen-catalyzed hydrolysis of the two substrates at its optimal catalytic condition. We showed that for semi-pro*Pb*PM4, the *K*_m_ values were much lower than the mature enzyme (<5 μM vs. >100 μM) such that the *k*_cat_ values were unable to be accurately determined.

The inhibition of two competitive compounds, pepstatin A and Ro40-4388, against mature *Pb*PM4 was assessed (Table 3). Similar to the FV plasmepsin orthologs of human malaria parasites [39, 40, 56, 63], *Pb*PM4 was strongly inhibited in sub-nanomolar magnitude by pepstatin A, a tight-binding inhibitor of the pepsin-like aspartic proteinases. Ro40-4388, a peptidomimetic inhibitor highly selective to *Pf*PM1 [40], inhibited *Pb*PM4 in the nanomolar range, comparable with its inhibition of *Pf*PM2 and *Pf*PM4 [63, 64].

**Table 3:** Kinetic analyses of the inhibition of *Pb*PM4.

Inhibitor^a^	*K*_i_ (nM)
pepstatin A	0.11 ± 0.02
Ro40-4388	135 ± 21

^a^The structures of the tested inhibitors are shown in S2 Fig.

### Subsite preferences

#### Primary subsite preferences

**Fig 5:**
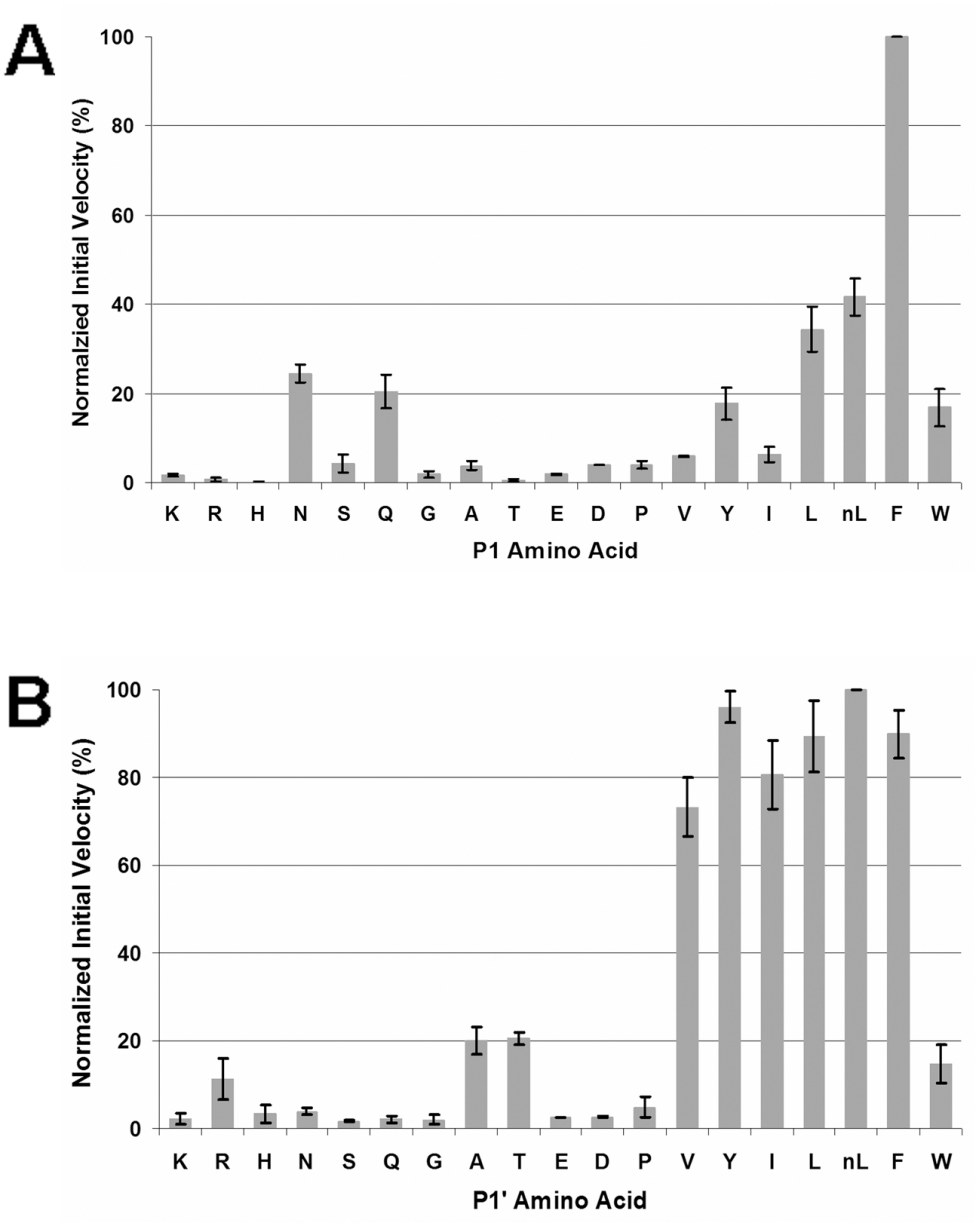
Primary subsite preferences of *Pb*PM4. The initial velocities for hydrolysis of the P1 (A) and P1’ (B) library pools were determined spectroscopically and normalized to the highest cleavage velocities, which were set to 100 percent. Phenylalanine, leucine, and norleucine were the three most favored amino acid substitutes at P1; whereas norleucine, tyrosine, and phenylalanine were the three most favored at P1’.

For the P1 library, phenylalanine was the most favorite amino acid substitute (Fig 5A). Two aliphatic residues, leucine and norleucine, were the second best substitutes in that the hydrolysis rates of the leucine and norleucine pools were 34% and 42% of that of the phenylalanine pool, respectively. In addition, no levels of hydrolysis were also detected for peptide pools containing four other P1 amino acid substitutes, asparagine (25%), glutamine (20%), tyrosine (18%) and tryptophan (17%). Hydrolyzed peptide products from the other 12 peptide pools, however, were barely detected.

The optimal P1’ amino acid substitutes were hydrophobic residues with norleucine the best (Fig 5B). Peptide pools containing the other aromatic or aliphatic P1’ substitutes, except for tryptophan, all had more than 70% of the hydrolysis rate of the P1’ norleucine pool. Notably, the initial rates of the peptide pools decreased as the size of their P1’ amino acid side chains reduced from leucine to glycine or expanded to tryptophan, which was possibly due to insufficient interactions or steric hindrance with residues at the S1’ subsite. In addition, similar to the results from the P1 library, *Pb*PM4 did not exhibit remarkable hydrolysis on the peptide pools containing most of the polar and charged P1’ residues.

#### Secondary subsite preferences

The best P1 and P1’ pools (i.e., the phenylalanine, norleucine and leucine pools for the P1 library; and the norleucine, tyrosine and phenylalanine pools for the P1’ library) were subject to LC/MS analysis.

**Fig 6:**
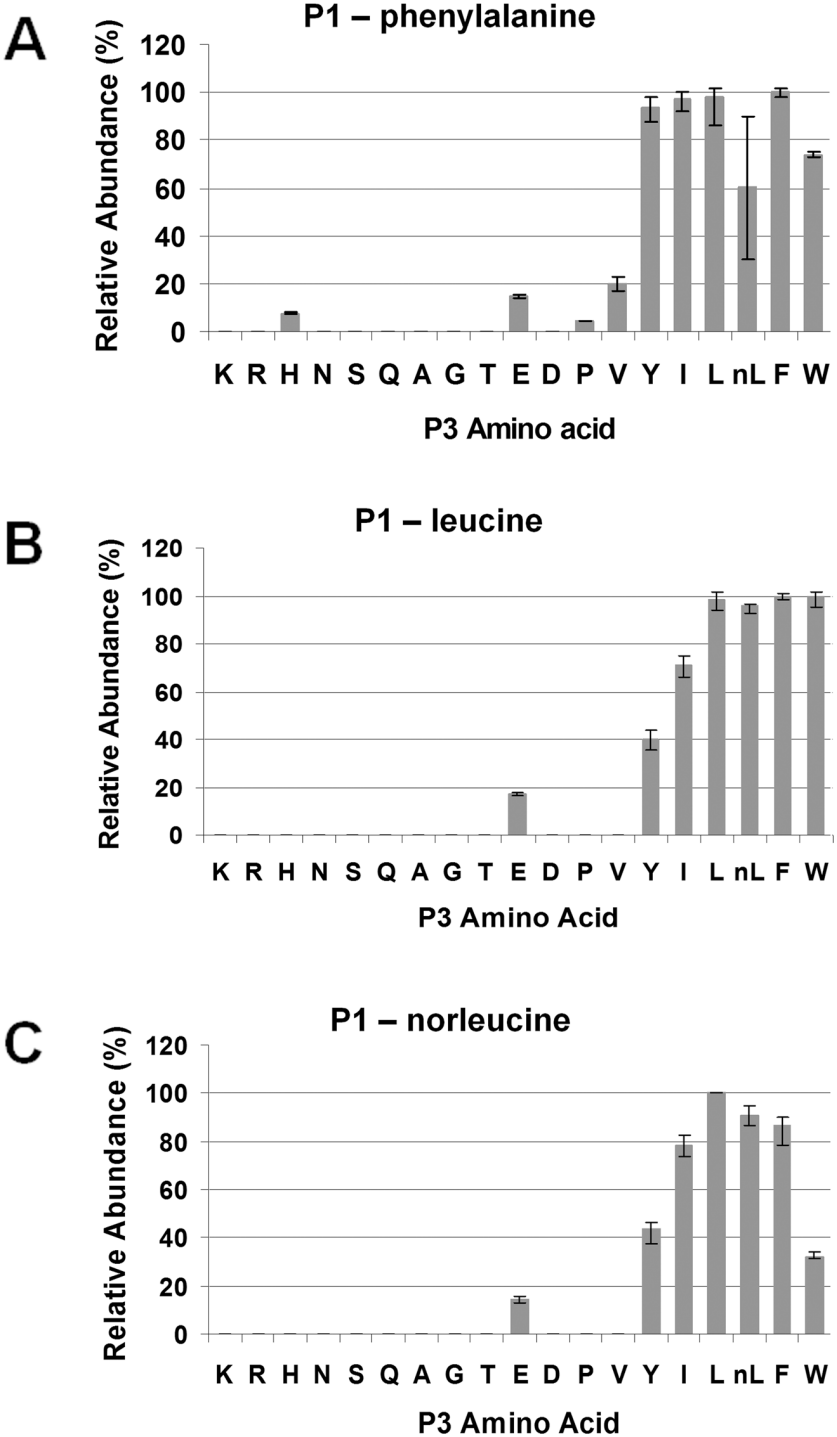
Secondary subsite preferences of *Pb*PM4 at S3. The three most favored peptide pools, P1-phenylalanine (A), P1-leucine (B) and P1-norleucine (C), were used for analyzing the S3 subsite preferences of *Pb*PM4. The relative abundances of penta-peptides varying at P3 were determined using in-line LC/MS, and normalized to the quantity of the most abundant cleavage product, which was set to 100 percent. The peptide abundances were plotted against the P3 amino acid substitutes.

For the S3 subsite, large hydrophobic residues were the favored in the P1- phenylalanine pool: digested penta-peptides containing phenylalanine, leucine, norleucine and isoleucine at P3 were the most abundant (Fig 6A), which was similar to the results for the P1-leucine and P1-norleucine pools (Fig 6B and 6C). P3-tryptophan was preferred in the phenylalanine and leucine pools, but not the P1-norleucine pool; whereas the P3-tyrosine-containing penta-peptides were only in high relative abundance in the phenylalanine pool. In contrast, polar and charged amino acids, except for glutamic acid, were unanimously unfavored at P3 in all three tested pools.

**Fig 7:**
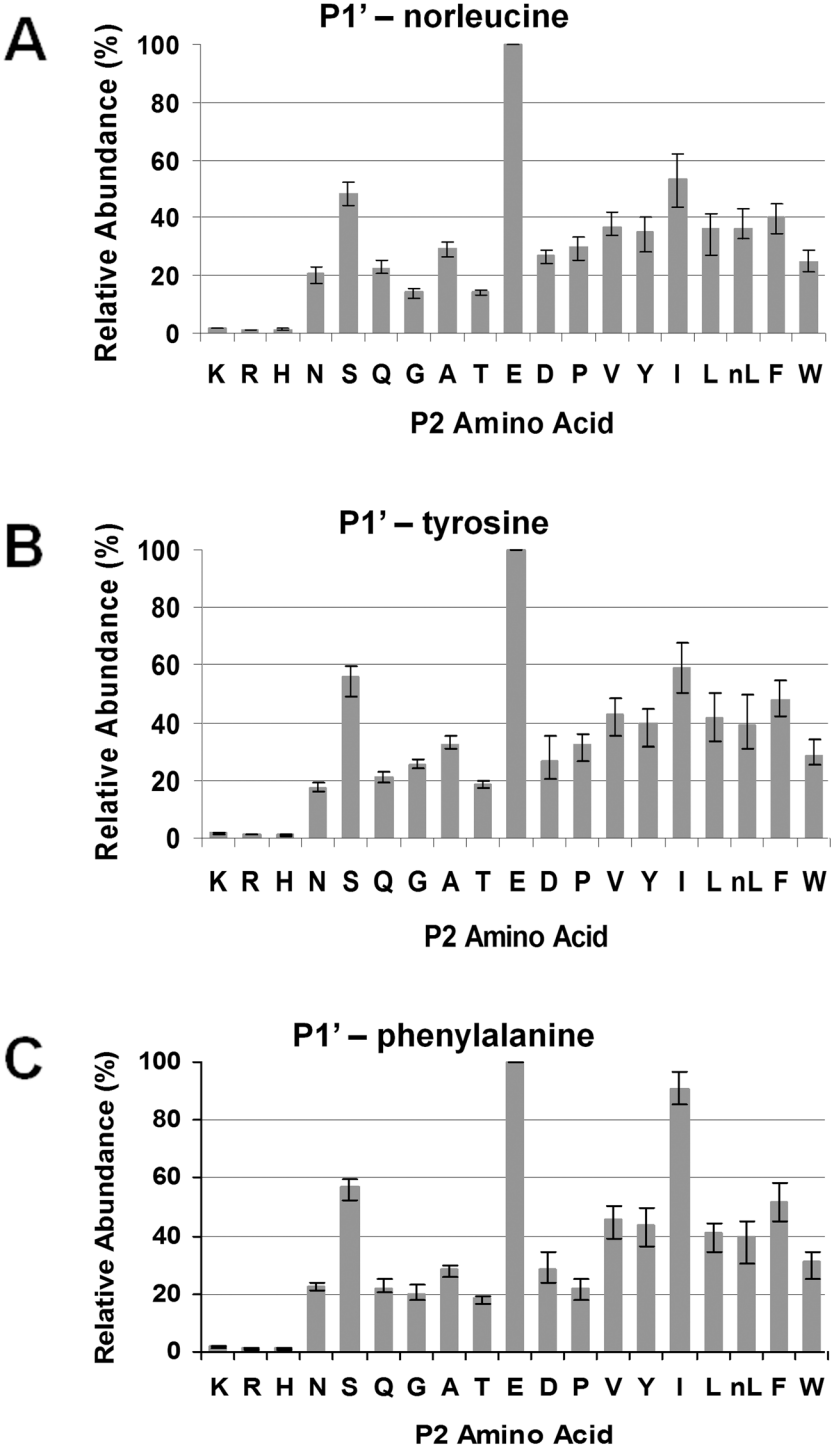
Secondary subsite preferences of *Pb*PM4 at S2. The three most favored peptide pools, P1-norleucine (A), P1-tyrosine (B) and P1-phenylalanine (C), were used for analyzing the S2 subsite preferences of *Pb*PM4. The relative abundances of penta-peptides varying at P2 were determined using in-line LC/MS, and normalized to the quantity of the most abundant cleavage product, which was set to 100 percent. The peptide abundances were plotted against the P2 amino acid substitutes.

While subsite S3 highly favored accommodation of hydrophobic residues, the S2 subsite was tolerant of P2 amino acids of different properties (Fig 7). For the P1’-norleucine pool, most of the substitutes led to at least 20% of the maximum abundance, except for the three basic residues, lysine, histidine, and arginine. Glutamic acid was best accommodated at S2, followed by isoleucine and serine. Similar results were found in the P1’-tyrosine and P1’-phenylalanine pools.

**Fig 8:**
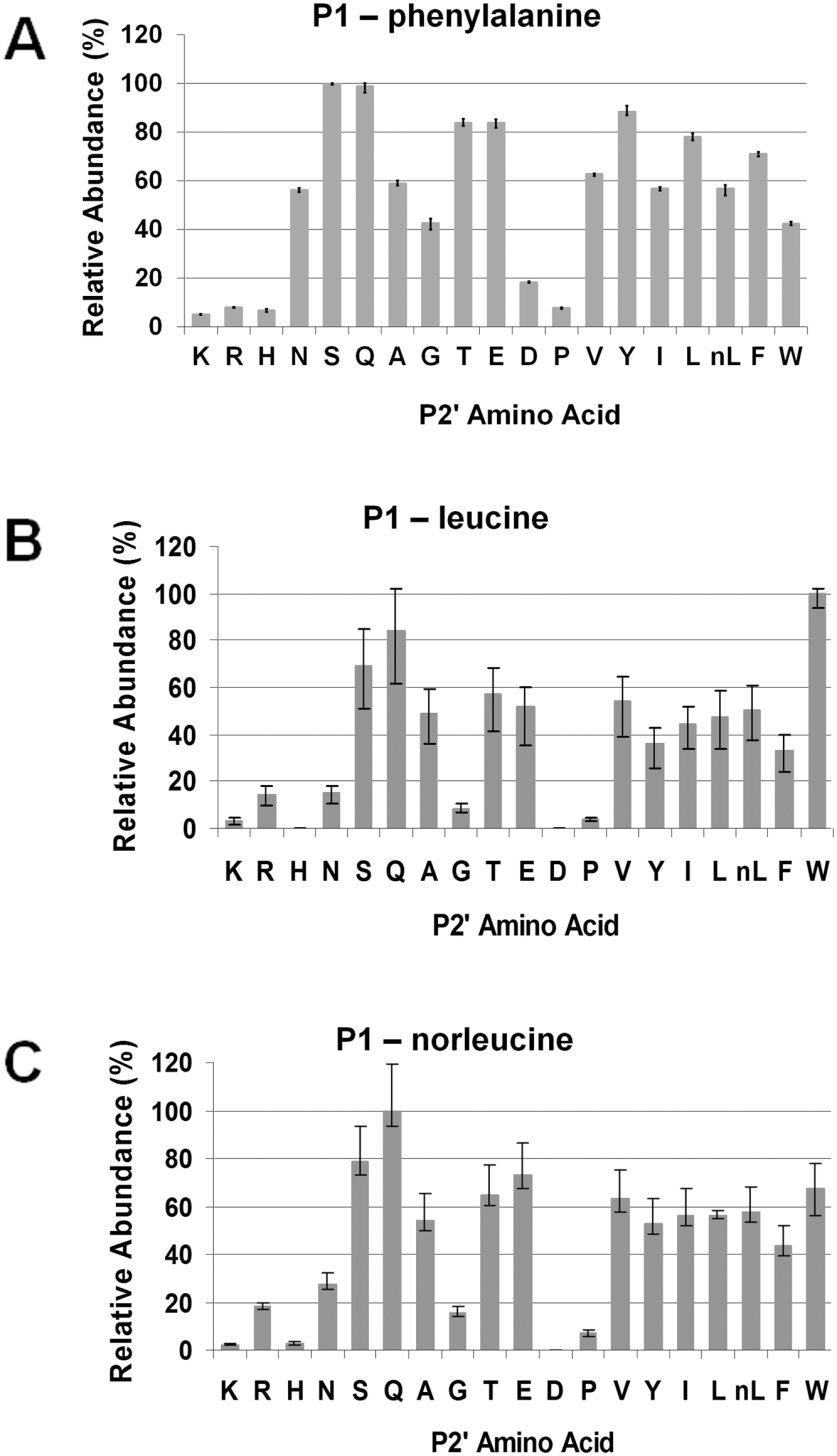
Secondary subsite preferences of *Pb*PM4 at S2’. The three most favored peptide pools, P1-phenylalanine (A), P1-leucine (B) and P1-norleucine (C), were used for analyzing the S2’ subsite preferences of *Pb*PM4. The relative abundances of tri-peptides varying at P2’ were determined using in-line LC/MS, and normalized to the quantity of the most abundant cleavage product, which was set to 100 percent. The peptide abundances were plotted against the P2’ amino acid substitutes.

Similarly, P2’ amino acid substitutes of varied properties were well accommodated in the S2’ subsite (Fig 8). For the P1-pheylalanine and P1- norleucine pools, serine and glutamine were the two most favored; whereas for the P1-leucine pool, tryptophan was best accommodated. Hydrophobic amino acids other than proline and hydrophilic ones, such as threonine and glutamic acid, were accommodated equally well at S2’. However, charged amino acids, such as aspartic acid, lysine, histidine, and arginine, were not favored.

**Fig 9:**
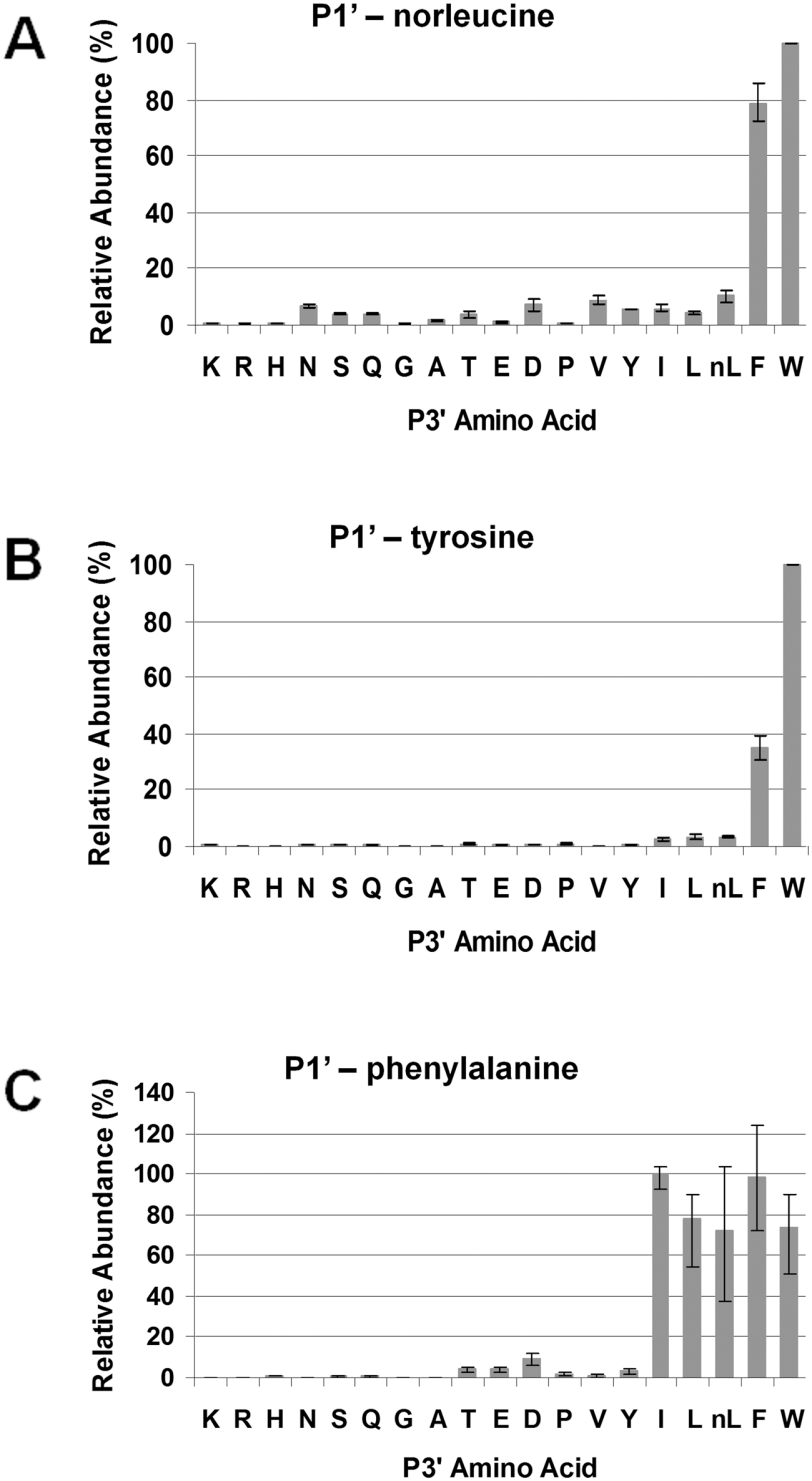
Secondary subsite preferences of *Pb*PM4 at S3’. The three most favored peptide pools, P1-norleucine (A), P1-tyrosine (B) and P1-phenylalanine (C), were used for analyzing the S3’ subsite preferences of *Pb*PM4. The relative abundances of tri-peptides varying at P3’ were determined using in-line LC/MS, and normalized to the quantity of the most abundant cleavage product, which was set to 100 percent. The peptide abundances were plotted against the P3’ amino acid substitutes.

*Pb*PM4 showed high selectivity to the P3’ substitutes in that only the aromatic residues tryptophan and phenylalanine were well accepted in the P1’-norleucine pool, and peptide cleavage products containing other P3’ amino acid substitutes were barely detected (Fig 9A). Similar results were also found in the P1’-phenylalanine and P1’-tyrosine pools (Fig 9B and 9C).

### Inhibition analysis

Compound 1 (KPYEFΨRQF, where Ψ = -CH2-NH-) was designed as a selective inhibitor of *Pb*PM4 versus hcatD based on findings of the subsite preference study and the rationale described in the peptidomimetic inhibitor design and inhibition analyses section of Materials and Methods. Similarly, compounds 2–7 were designed as selective inhibitors of *Pf*PM1, *Pf*PM2, *Pf*PM4, *Pv*PM4, *Po*PM4 and *Pm*PM4, respectively. As an example, the original data showing primary and secondary subsite preferences of *Po*PM4 and hcatD (Tables G-L in S1 File) were reported to help better understand the rationale for the design of compound 6. The dissociation constants (*K*_i_) of compounds 1–7 were determined for *Pb*PM4 (Table 4). Meanwhile, the *K*_i_ values of the newly developed compound 1 on six FV plasmepsins of human malaria parasites and hcatD were determined.

**Table 4:** The inhibition of peptidomimetic compounds on FV plasmepsins and hcatD.

		Dissociation Constant (*K*_i_) (nM)							
Compound	Sequence^a^	*Pb*PM4	*Pf*PM1	*Pf*PM2	*Pf*PM4	*Pv*PM4^b^	*Po*PM4^b^	*Pm*PM4^b^	hCatD
1	KPYEFΨRQF	3,900 ± 400	> 100,000	35,000 ± 4,000	190 ± 19	426 ± 44	1,900 ± 90	445 ± 44	41,000 ± 4,000
2	KPFSLΨLQF	379 ± 26	43 ± 5^d^	271 ± 26^d^	209 ± 18^d^	101 ± 11^d^	767 ± 81^d^	272 ± 20^d^	232 ± 14^d^
3	KPnLSnLΨLQI	375 ± 54	72.8 ± 8.8^d^	13.9 ± 1.8^c^	21.7 ± 2.7^c^	97 ± 14^c^	187 ± 29^c^	160 ± 26^c^	219 ± 21^c^
4	KPVEFΨRQT	502 ± 56	31,000 ± 5,000^d^	>20,000^c^	2.4 ± 0.3^c^	14.4 ± 2.1^c^	39.0 ± 4.5^c^	10.3 ± 1.3^c^	30.4 ± 2.0^c^
5	KPLEFΨFRV	1.4 ± 0.1	5,500 ± 700^d^	4,300 ± 800^c^	0.085 ± 0.014^c^	0.582 ± 0.084^c^	3.2 ± 0.5^c^	3.7 ± 0.6^c^	4.7 ± 0.4^c^
6	KPLEFΨYRV	0.12 ± 0.031	38,000 ± 6,000^d^	19,500 ± 4,000^c^	0.476 ± 0.087^c^	0.684 ± 0.087^c^	3.2 ± 0.5^c^	0.342 ± 0.047^c^	8.5 ± 0.6^c^
7	KPFELΨAWT	8,100 ± 800	42,000 ± 7,000^d^	16,600 ± 3,100^c^	12,700 ± 1,600^c^	9,800 ± 1,800^c^	> 20,000^c^	9,000 ± 1,200^c^	12,700 ± 1,200^c^

^a^Ψ = -CH2-NH-, nL = norleucine.

^b^ *Pf*PM1, *Pf*PM2 and *Pf*PM4, plasmepsins 1, 2 and 4 from *Plasmodium falciparum*, respectively; *Pv*PM4, plasmepsin 4 from *P*. *vivax*; *Po*PM4, plasmepsin 4 from *P*. *ovalae*; and *Pm*PM4, plasmepsin 4 from *P*. *malariae*.

^c^These dissociation constants were reported in [41].

^d^These dissociation constants were reported in [42].

Compounds 1–7 showed a wide range of inhibition of *Pb*PM4, with *K*_i_ values from picomolar to micromolar in magnitude. Compound 1 selectively inhibited *Pb*PM4 by a factor of more than 10-fold over hcatD and showed a binding affinity in the micromolar range. Compound 6, however, bound tightly to *Pb*PM4 in sub-nanomolar magnitude, and showed more than 70-fold selectivity against hcatD, and therefore is the most selective peptidomimetic inhibitor of *Pb*PM4.

Interestingly, compound 1, designed as a selective inhibitor of *Pb*PM4, exhibited higher binding affinities to the plasmepsin 4 orthologs than to *Pf*PM1, *Pf*PM2 and hcatD. Also, most of the *K*_i_ values for *Pb*PM4 were in the same order of magnitude as those of its plasmepsin 4 orthologs from human malaria parasites. These observations indicate that plasmepsins 4 orthologs might share similar active site structures.

## Discussion

In this study, we reported the cloning, expression and enzymatic characterization of a recombinant form of the FV plasmepsin 4 from the rodent malaria parasite *P*. *berghei*. We showed that *Pb*PM4 is catalytically active even in the presence of the pro-segment. For the mature enzyme, we determined optimal catalytic conditions, studied the interaction with generic substrates, and assayed inhibitors of pepsin-like aspartic proteinases. We then employed combinatorial peptide libraries to explore the subsite preferences of *Pb*PM4 and developed a peptidomimetic inhibitor, which, upon inhibition analysis, showed micromolar binding affinity to *Pb*PM4 and more than 10-fold selectivity to *Pb*PM4 over hcatD. Findings of our study provide knowledge on the active site preferences of *Pb*PM4 and plasmepsin inhibitor design.

The recombinant semi-pro*Pb*PM4 is able to perform catalysis in the presence of the N-terminal pro-segment. This enzymatic activity has not been shown in any other FV plasmepsin studied so far. As for the non-FV plasmepsins, Xiao *et al*. reported that plasmepsin 5 (*Pf*PM5) of *P*. *falciparum*, a membrane protein involving in exporting effector proteins of parasite to human red blood cells [26, 27], shows a similar phenomenon [65]. Also, a similar finding has been reported in renin, an aspartic proteinase playing essential roles in the regulation of blood pressure and electrolyte balance: inactive renin zymogen gains full catalytic activity without pro-segment processing due to a potential conformational change when dialyzed against acidic buffer of pH 3.3 at 10°C [66]. However, unlike the cases of *Pf*PM5 and renin, where the non-proteolytically activated zymogen kinetically resembles the mature enzyme [65, 66], *Pb*PM4, in the presence of the truncated pro-segment, has *K*_m_ values of peptide substrates two orders of magnitude greater than those for the converted mature enzyme. This may be due to the competitive binding of the N-terminal flexible segment to the active site cleft, or because the presence of the pro-segment leads the active site cleft to conformations that do not allow proper accommodation of substrates, as in the case of semi-pro*Pf*PM2 [67]. Another aspartic proteinase that shares this feature is β-secretase (BACE), where the pro-segment does not suppress enzyme activity but appears to facilitate proper folding of the active proteinase domain [68].

Our findings do not agree with a previous report showing that the recombinant wild-type pro*Pb*PM4 was unable to conduct auto-maturation to gain catalytic activity [69]. This controversy may arise from different refolding and/or purification approaches employed. In particular, we found that refolded protein prior to size exclusion chromatography purification was catalytically active, but was unable to perform auto-maturation; however, when separated from a majority of misfolded protein, which showed neither auto-maturation nor activity, the rest gained auto-maturation capability while maintaining catalytic activity (Figs 2–4). Therefore, it seems that the misfolded protein masks the authentic auto-maturation of wild-type pro*Pb*PM4.

The naturally-occurring form of mature *Pf*PM2 has been recombinantly expressed in *E*. *coli*, refolded and purified. The resulting enzyme exhibits comparable catalytic efficiency (*k*_cat_/*K*_m_) to the *in vitro* auto-matured product of *Pf*PM2 zymogen, which still retains 14 extra residues from its N-terminal pro-segment [70]. To understand whether catalytically active, recombinant mature *Pb*PM4 can be obtained without the presence of the pro-segment, we cloned the sequence encoding solely the C-terminal 326 amino acid residues. Expression of the protein in *E*. *coli* failed as no detec level of *Pb*PM4 was obtained, possibly due to intra- and/or inter-molecular degradation of the refolded mature enzyme. This may indicate that the N-terminal pro-segment of *Pb*PM4 plays a critical role in stabilizing the mature enzyme in addition to guiding proper folding.

The subsite preferences of *Pb*PM4 are compared with those of other plasmepsins (*Pf*PM1, *Pf*PM2, *Pf*PM4, *Pv*PM4, *Po*PM4 and *Pm*PM4) and human aspartic proteinase homologs (pepsin A and cathepsins D and E) from previous studies using the same libraries [41, 42, 71].

The primary subsite preferences of *Pb*PM4 reveal high consistency with those of the other enzymes: the S1 subsites in nine of the ten enzymes accommodate phenylalanine best; the S1’ subsites also favor bulky hydrophobic side chains, though the optimal substitutes are shared by five distinct amino acids—phenylalanine, leucine, tyrosine, isoleucine, and norleucine. This is not surprising, since residues comprised of these two subsites are highly conserved among these proteinases (Table M in S1 File).

Hydrophobic amino acids are consistently favored at the S3 and S3’ subsites among the ten aspartic proteinases investigated. In the P3 position, the best substitutes for all tested enzymes are restricted to the three aliphatic residues, isoleucine, leucine, and norleucine, and the three aromatic residues, tyrosine, phenylalanine, and tryptophan; and similar findings are observed in the P3’ position. Residues comprising the S3 subsite are generally not conserved among FV plasmepsins and human aspartic proteinase homologs, except Phe117, which may play a critical role in interacting with side chains of the P3 amino acids. Unlike the other nine proteinases, *Pb*PM4 employs three serine residues to constitute half the S3 subsite. Understanding how hydrophobic amino acids are accommodated in such a polar residue-enriched pocket will require future structural studies. As for the residue composition of the S3’ subsite, plasmepsin 4 orthologs share identical residues, which are quite distinct from the three human orthologs as well as *Pf*PM1 and *Pf*PM2. Despite the different composition, all the ten enzymes share a similar amino acid preference profile. Notably, P3-tyrosine and P3-phenylalanine are comparably favored in all tested enzymes except for *Pb*PM4, where P3-phenylalanine (99%) is overwhelmingly preferred over P3-tyrosine (3%).

Unlike the strong preferences exhibited in the S3 and S3’ subsites, the amino acids that are favored specificities at S2 and S2’ are more widespread in nature. For example, charged amino acids such as P2-glutamic acid, and polar amino acids such as P2’-serine and P2’-glutamine, are well accepted by both the human enzyme and the plasmepsins. Residues comprising the S2 subsites are well conserved among these enzymes, especially among the plasmepsin 4 orthologs. As a result, the three best amino acid substitutes for the P2 position of the plasmepsin 4 enzymes are consistently glutamic acid, isoleucine, and serine; indeed, P2-isoleucine and P2-serine are overall the most favored among the ten studied enzymes. In contrast, residues comprising the S2’ subsites, particularly residue 74, are poorly conserved among the ten enzymes. The results indicate that human aspartic proteinases prefer accepting hydrophobic amino acids, whereas glutamine is readily accepted by most plasmepsins.

It is striking to see that compound 1, a composition of the amino acids that are favored by *Pb*PM4 over hcatD, exhibits a decent selection of the target enzyme over the human counterpart; after all, numerous enzymes including plasmepsins [72–75], upon substrate or inhibitor binding, adopt the “induced-fit” [76] or “conformational ensembles”, [77] rather than the “lock-and-key” mechanism [78]. Nonetheless, compound 6, a *Po*PM4 inhibitor designed to be specific over hcatD, turns out to be the one binding more strongly to, and with a higher selectivity to *Pb*PM4 over the human enzyme, indicating a coordinative effect of varied functional groups within a compound on determining its enzyme-binding properties.

Though pepstatin A and Ro40-4388 inhibit growth of *P*. *falciparum* and tightly bind multiple FV plasmepsins of human malaria parasites, they are not selective plasmepsin inhibitors [40, 63, 79, 80]. For the past 25 years, various types of peptidomimetic, non-peptidic and bi-functional compounds have been screened for possible inhibitors targeting FV plasmepsins based on criteria such as inhibition potency to plasmepsins, binding selectivity to plasmepsins over their human proteinase homologs, growth inhibition of cultured malaria parasites and cytotoxicity to mammalian cell culture [80–82]. Aside from this study, there were other investigations in which the inhibition of compounds was analyzed on multiple FV plasmepsins. For example, Nöteberg and colleagues showed that certain hydroxyethylamine derivatives inhibit *Pf*PM1, 2 and 4 in nanomolar magnitude, have a >30-fold binding selectivity over hcatD and block growth of cultured *P*. *falciparum* with IC_50_ values in the low micromolar range [81, 83, 84]. Nezami and colleagues found that several allophenylnorstatine-based compounds inhibit all four FV plasmepsins of *P*. *falciparum* in nanomolar magnitude and block parasite growth with IC_50_ values also in the low micromolar range [81, 85, 86]. These compounds were later modified with the TD_50_ (cytotoxicity) improved to be in the high micromolar range to rat skeletal myoblasts [87]. In addition, Skinner-Adams, Hobbs and colleagues reported that clinically utilized human immunodeficiency virus (HIV) protease inhibitors exhibit anti-malarial activity on parasites at both erythrocytic and pre-erythrocytic stages [88–90] and inhibit *Pf*PM2 and *Pf*PM4 [91]. Interestingly, using affinity binding probes coupled to an FV plasmepsin inhibitor library, Liu *et al*. identified a hydroxyethyl-based inhibitor that inhibits all four FV plasmepsins and the growth of cultured *P*. *falciparum* with IC_50_ at ~1 μM [92].

Despite all the efforts on drug development, the role of FV plasmepsins in malaria pathogenesis is still not fully understood. Genetic ablation of all four FV plasmepsin genes leads to a decreased growth rate and abnormal FV structures of cultured *P*. *falciparum*, which nonetheless survive [93]. These findings suggest that the function of FV plasmepsins may be dispensable. If so, what are the molecular targets of those FV plasmepsin inhibitors that show anti-malarial activity? Independent studies from different laboratories showed a comparable growth sensitivity between the parent line and FV plasmepsin-KO mutants in the presence of inhibitors such as pepstatin A, Ro40-4388, HIV protease inhibitors, hydroxyethylamine-based inhibitors, 1,2-dihydroxyethylene derivatives and diphenylurea compounds [79, 93–95], thus suggesting that the FV plasmepsins are not the primary targets for these tested compounds to exhibit anti-malarial activity. Instead, a growing body of evidence has indicated that non-FV plasmepsins, such as plasmepsins 5 and 10 may be the primary targets of certain aspartic proteinase inhibitors. For example, over-expression or knockdown of *Pf*PM5 affects parasite sensitivity to a transition-state peptidomimetic inhibitor [96] and over-expression of *Pf*PM10, a protein with unknown function, decreases the inhibition potency of a diphenylurea-derived compound to the growth of cultured parasite [79].

Selective inhibitors of plasmepsin 5 versus human aspartic proteinase homologs have been developed and shown inhibition potency to parasite growth [97]. However, the specificity of these compounds and their possible inhibition of FV plasmepsins are not yet known. It is also unclear whether FV plasmepsins are also targeted inside the parasite by these plasmepsin 5 inhibitors, and if so, how inhibition of FV plasmepsins contributes to the overall anti-malarial effects. These questions need to be addressed in future development of anti-malarial drugs targeting plasmepsins.

## Supporting Information

S1 Fig Primary structure of pro*Pb*PM4. (TIF)

S2 Fig Structures of inhibitors tested in Table 3. (TIF)

S1 File Supporting tables and references. This file contains the following items: 1) Table A Comparison of primary subsite preferences of *Pb*PM4 and hcatD at S1, 2) Table B Comparison of primary subsite preferences of *Pb*PM4 and hcatD at S1’, 3) Table C Comparison of secondary subsite preferences of *Pb*PM4 and hcatD at S3, 4) Table D Comparison of secondary subsite preferences of *Pb*PM4 and hcatD at S2, 5) Table E Comparison of secondary subsite preferences of *Pb*PM4 and hcatD at S2’, 6) Table F Comparison of secondary subsite preferences of *Pb*PM4 and hcatD at S3’, 7) Table G Comparison of primary subsite preferences of *Po*PM4 and hcatD at S1, 8) Table H Comparison of primary subsite preferences of *Po*PM4 and hcatD at S1’, 9) Table I Comparison of secondary subsite preferences of *Po*PM4 and hcatD at S3, 10) Table J Comparison of secondary subsite preferences of *Po*PM4 and hcatD at S2, 11) Table K Comparison of secondary subsite preferences of *Po*PM4 and hcatD at S2’, 12) Table L Comparison of secondary subsite preferences of *Po*PM4 and hcatD at S3’, 13) Table M Amino acid residues that constitute the S3-S3’ subsite pockets of human and malaria aspartic proteinases, 14) References A.
(DOC)

## References

[1] Kendrick RK. Taxonomy, Zoogeography and Evolution In: Kendrick RK, Peters W, editors. Rodent Malaria. London New York San Francisco: Academic Press; 1978 p. 1–52.

[2] Waters AP, Higgins DG, McCutchan TF. Plasmodium falciparum appears to have arisen as a result of lateral transfer between avian and human hosts. Proceedings of the National Academy of Sciences of the United States of America. 1991;88(8):3140–4. 2014232 10.1073/pnas.88.8.3140 PMC51401

[3] Landau I, Boulard Y. Life Cycles and Morphology In: Kendrick RK, Peters W, editors. Rodent Malaria. London New York San Francisco: Academic Press; 1978 p. 53–84.

[4] Sinden RE. Cell Biology In: Kendrick RK, Peters W, editors. Rodent Malaria. London New York San Francisco: Academic Press; 1978 p. 85–169.

[5] Aikawa M, Seed TM. Morphology of plasmodia In: Kreier JP, editor. Malaria. 1. New York: Academic Press; 1980 p. 285–344.

[6] Janse CJ, Mons B, Croon JJ, van der Kaay HJ. Long-term in vitro cultures of Plasmodium berghei and preliminary observations on gametocytogenesis. International journal for parasitology. 1984;14(3):317–20. . 6381348 10.1016/0020-7519(84)90083-3

[7] Janse CJ, Boorsma EG, Ramesar J, Grobbee MJ, Mons B. Host cell specificity and schizogony of Plasmodium berghei under different in vitro conditions. International journal for parasitology 1989 p. 509–14. 2674047 10.1016/0020-7519(89)90080-5

[8] Trager W, Jensen JB. Human malaria parasites in continuous culture. Science. 1976;193(4254):673–5. . 781840 10.1126/science.781840

[9] Trager W, Jensen JB. Cultivation of erythrocytic stages. Bulletin of the World Health Organization. 1977;55(2–3):363–5. 338187 PMC2366733

[10] Goodman AL, Forbes EK, Williams AR, Douglas AD, de Cassan SC, Bauza K, et al The utility of Plasmodium berghei as a rodent model for anti-merozoite malaria vaccine assessment Sci Rep-Uk. 2013;3 Artn 1706 10.1038/Srep01706 WOS:000317892500009. PMC3632886 23609325

[11] Vanvianen PH, Klayman DL, Lin AJ, Lugt CB, Vanengen AL, Vanderkaay HJ, et al Plasmodium-Berghei—the Antimalarial Action of Artemisinin and Sodium Artelinate Invivo and Invitro, Studied by Flow-Cytometry. Experimental parasitology. 1990;70(2):115–23. 10.1016/0014-4894(90)90092-Q WOS:A1990CM71600001. 2404778

[12] Mlambo G, Kumar N. Transgenic Rodent Plasmodium berghei Parasites as Tools for Assessment of Functional Immunogenicity and Optimization of Human Malaria Vaccines. Eukaryot Cell. 2008;7(11):1875–9. 10.1128/Ec.00242-08 WOS:000260623300001. 18806208 PMC2583535

[13] Adepiti AO, Elujoba AA, Bolaji OO. In vivo antimalarial evaluation of MAMA decoction on Plasmodium berghei in mice. Parasitol Res. 2014;113(2):505–11. 10.1007/s00436-013-3680-0 WOS:000333028100007. 24271081

[14] Musila MF, Dossaji SF, Nguta JM, Lukhoba CW, Munyao JM. In vivo antimalarial activity, toxicity and phytochemical screening of selected antimalarial plants. J Ethnopharmacol. 2013;146(2):557–61. 10.1016/j.jep.2013.01.023 WOS:000316976400015. 23376043

[15] Orjuela-Sanchez P, Duggan E, Nolan J, Frangos JA, Carvalho LJM. A lactate dehydrogenase ELISA-based assay for the in vitro determination of Plasmodium berghei sensitivity to anti-malarial drugs. Malaria J. 2012;11 Artn 366 10.1186/1475-2875-11-366 WOS:000313239900001. PMC3538577 23126583

[16] Boniface PK, Pal A. Substantiation of the ethnopharmacological use of Conyza sumatrensis (Retz.) E.H.Walker in the treatment of malaria through in-vivo evaluation in Plasmodium berghei infected mice. J Ethnopharmacol. 2013;145(1):373–7. 10.1016/j.jep.2012.10.025 WOS:000313604800046. 23123263

[17] Lin JW, Annoura T, Sajid M, Chevalley-Maurel S, Ramesar J, Klop O, et al A Novel ' Gene Insertion/Marker Out' (GIMO) Method for Transgene Expression and Gene Complementation in Rodent Malaria Parasites. Plos One. 2011;6(12). ARTN e29289 WOS:000300674900025. 10.1371/journal.pone.0029289 PMC3246482 22216235

[18] Mesfin A, Giday M, Animut A, Teklehaymanot T. Ethnobotanical study of antimalarial plants in Shinile District, Somali Region, Ethiopia, and in vivo evaluation of selected ones against Plasmodium berghei. J Ethnopharmacol. 2012;139(1):221–7. 10.1016/j.jep.2011.11.006 WOS:000299976900030. 22101085

[19] Kamiyama T, Matsubara J. Application of a Simple Culture of Plasmodium-Berghei for Assessment of Antiparasitic Activity. International journal for parasitology. 1992;22(8):1137–42. WOS:A1992KE72700007. 1487372 10.1016/0020-7519(92)90032-g

[20] Li J, Zhu JD, Appiah A, Mccutchan TF, Long GW, Milhous WK, et al Plasmodium-Berghei—Quantitation of Invitro Effects of Antimalarial-Drugs on Exoerythrocytic Development by a Ribosomal-Rna Probe. Experimental parasitology. 1991;72(4):450–8. 10.1016/0014-4894(91)90091-A WOS:A1991FK85500013. 2026219

[21] Coombs GH, Goldberg DE, Klemba M, Berry C, Kay J, Mottram JC. Aspartic proteases of Plasmodium falciparum and other parasitic protozoa as drug targets. Trends in parasitology. 2001;17(11):532–7. . 11872398 10.1016/s1471-4922(01)02037-2

[22] Banerjee R, Liu J, Beatty W, Pelosof L, Klemba M, Goldberg DE. Four plasmepsins are active in the Plasmodium falciparum food vacuole, including a protease with an active-site histidine. Proceedings of the National Academy of Sciences of the United States of America. 2002;99(2):990–5. 10.1073/pnas.022630099 11782538 PMC117418

[23] Bozdech Z, Llinas M, Pulliam BL, Wong ED, Zhu J, DeRisi JL. The transcriptome of the intraerythrocytic developmental cycle of Plasmodium falciparum. PLoS biology. 2003;1(1):E5 10.1371/journal.pbio.0000005 12929205 PMC176545

[24] Gardner MJ, Hall N, Fung E, White O, Berriman M, Hyman RW, et al Genome sequence of the human malaria parasite Plasmodium falciparum. Nature. 2002;419(6906):498–511. 10.1038/nature01097 12368864 PMC3836256

[25] Hall N, Pain A, Berriman M, Churcher C, Harris B, Harris D, et al Sequence of Plasmodium falciparum chromosomes 1, 3–9 and 13. Nature. 2002;419(6906):527–31. 10.1038/nature01095 . 12368867

[26] Boddey JA, Hodder AN, Gunther S, Gilson PR, Patsiouras H, Kapp EA, et al An aspartyl protease directs malaria effector proteins to the host cell. Nature. 2010;463(7281):627–31. 10.1038/nature08728 20130643 PMC2818761

[27] Russo I, Babbitt S, Muralidharan V, Butler T, Oksman A, Goldberg DE. Plasmepsin V licenses Plasmodium proteins for export into the host erythrocyte. Nature. 2010;463(7281):632–6. 10.1038/nature08726 20130644 PMC2826791

[28] Mastan BS, Kumari A, Gupta D, Mishra S, Kumar KA. Gene disruption reveals a dispensable role for plasmepsin VII in the Plasmodium berghei life cycle. Molecular and biochemical parasitology. 2014;195(1):10–3. 10.1016/j.molbiopara.2014.05.004 . 24893340

[29] Carlton JM, Galinski MR, Barnwell JW, Dame JB. Karyotype and synteny among the chromosomes of all four species of human malaria parasite. Molecular and biochemical parasitology. 1999;101(1–2):23–32. . 10413040 10.1016/s0166-6851(99)00045-6

[30] Dame JB, Yowell CA, Omara-Opyene L, Carlton JM, Cooper RA, Li T. Plasmepsin 4, the food vacuole aspartic proteinase found in all Plasmodium spp. infecting man. Molecular and biochemical parasitology. 2003;130(1):1–12. . 14550891 10.1016/s0166-6851(03)00137-3

[31] Sherman IW. Amino acid metabolism and protein synthesis in malarial parasites. Bulletin of the World Health Organization. 1977;55(2–3):265–76. 338183 PMC2366754

[32] Naughton JA, Nasizadeh S, Bell A. Downstream effects of haemoglobinase inhibition in Plasmodium falciparum-infected erythrocytes. Molecular and biochemical parasitology. 2010;173(2):81–7. 10.1016/j.molbiopara.2010.05.007 . 20478341

[33] Lew VL, Tiffert T, Ginsburg H. Excess hemoglobin digestion and the osmotic stability of Plasmodium falciparum-infected red blood cells. Blood. 2003;101(10):4189–94. 10.1182/blood-2002-08-2654 . 12531811

[34] Krugliak M, Zhang J, Ginsburg H. Intraerythrocytic Plasmodium falciparum utilizes only a fraction of the amino acids derived from the digestion of host cell cytosol for the biosynthesis of its proteins. Molecular and biochemical parasitology. 2002;119(2):249–56. . 11814576 10.1016/s0166-6851(01)00427-3

[35] Spaccapelo R, Janse CJ, Caterbi S, Franke-Fayard B, Bonilla JA, Syphard LM, et al Plasmepsin 4-deficient Plasmodium berghei are virulence attenuated and induce protective immunity against experimental malaria. The American journal of pathology. 2010;176(1):205–17. 10.2353/ajpath.2010.090504 20019192 PMC2797883

[36] Spaccapelo R, Aime E, Caterbi S, Arcidiacono P, Capuccini B, Di Cristina M, et al Disruption of plasmepsin-4 and merozoites surface protein-7 genes in Plasmodium berghei induces combined virulence-attenuated phenotype. Sci Rep. 2011;1:39 10.1038/srep00039 22355558 PMC3216526

[37] Rodrigues-Duarte L, de Moraes LV, Barboza R, Marinho CR, Franke-Fayard B, Janse CJ, et al Distinct placental malaria pathology caused by different Plasmodium berghei lines that fail to induce cerebral malaria in the C57BL/6 mouse. Malar J. 2012;11:231 10.1186/1475-2875-11-231 22799533 PMC3485172

[38] Dame JB, Reddy GR, Yowell CA, Dunn BM, Kay J, Berry C. Sequence, expression and modeled structure of an aspartic proteinase from the human malaria parasite Plasmodium falciparum. Molecular and biochemical parasitology. 1994;64(2):177–90. . 7935597 10.1016/0166-6851(94)90024-8

[39] Luker KE, Francis SE, Gluzman IY, Goldberg DE. Kinetic analysis of plasmepsins I and II aspartic proteases of the Plasmodium falciparum digestive vacuole. Molecular and biochemical parasitology. 1996;79(1):71–8. . 8844673 10.1016/0166-6851(96)02651-5

[40] Moon RP, Tyas L, Certa U, Rupp K, Bur D, Jacquet C, et al Expression and characterisation of plasmepsin I from Plasmodium falciparum. European journal of biochemistry / FEBS. 1997;244(2):552–60. . 9119023 10.1111/j.1432-1033.1997.00552.x

[41] Beyer BB, Johnson JV, Chung AY, Li T, Madabushi A, Agbandje-McKenna M, et al Active-site specificity of digestive aspartic peptidases from the four species of Plasmodium that infect humans using chromogenic combinatorial peptide libraries. Biochemistry. 2005;44(6):1768–79. 10.1021/bi047886u . 15697202

[42] Liu P, Marzahn MR, Robbins AH, Gutierrez-de-Teran H, Rodriguez D, McClung SH, et al Recombinant plasmepsin 1 from the human malaria parasite plasmodium falciparum: enzymatic characterization, active site inhibitor design, and structural analysis. Biochemistry. 2009;48(19):4086–99. 10.1021/bi802059r 19271776 PMC2730762

[43] Westling J, Yowell CA, Majer P, Erickson JW, Dame JB, Dunn BM. Plasmodium falciparum, P. vivax, and P. malariae: a comparison of the active site properties of plasmepsins cloned and expressed from three different species of the malaria parasite. Experimental parasitology. 1997;87(3):185–93. 10.1006/expr.1997.4225 . 9371083

[44] Westling J, Cipullo P, Hung SH, Saft H, Dame JB, Dunn BM. Active site specificity of plasmepsin II. Protein science: a publication of the Protein Society. 1999;8(10):2001–9. 10.1110/ps.8.10.2001 10548045 PMC2144121

[45] Edman P. A method for the determination of amino acid sequence in peptides. Archives of biochemistry. 1949;22(3):475 . 18134557

[46] Moore S, Stein WH. Photometric ninhydrin method for use in the chromatography of amino acids. The Journal of biological chemistry. 1948;176(1):367–88. . 18886175

[47] Dunn BM, Scarborough PE, Davenport R, Swietnicki W. Analysis of proteinase specificity by studies of peptide substrates. The use of UV and fluorescence spectroscopy to quantitate rates of enzymatic cleavage. Methods in molecular biology. 1994;36:225–43. 10.1385/0-89603-274-4:225 . 7697110

[48] Scarborough PE, Guruprasad K, Topham C, Richo GR, Conner GE, Blundell TL, et al Exploration of subsite binding specificity of human cathepsin D through kinetics and rule-based molecular modeling. Protein science: a publication of the Protein Society. 1993;2(2):264–76. 10.1002/pro.5560020215 8443603 PMC2142340

[49] Marquardt DW. An algorithm for least-squares estimation of nonlinear parameters. J Soc Indust Appl Math. 1963;11(2):431–41.

[50] Henderson PJ. A linear equation that describes the steady-state kinetics of enzymes and subcellular particles interacting with tightly bound inhibitors. The Biochemical journal. 1972;127(2):321–33. 4263188 10.1042/bj1270321 PMC1178592

[51] Leatherbarrow RJ, Fersht AR, Winter G. Transition-state stabilization in the mechanism of tyrosyl-tRNA synthetase revealed by protein engineering. Proceedings of the National Academy of Sciences of the United States of America. 1985;82(23):7840–4. 3865201 10.1073/pnas.82.23.7840 PMC390865

[52] Morrison JF. Kinetics of the reversible inhibition of enzyme-catalysed reactions by tight-binding inhibitors. Biochimica et biophysica acta. 1969;185(2):269–86. . 4980133 10.1016/0005-2744(69)90420-3

[53] Dunn BM, Jimenez M, Parten BF, Valler MJ, Rolph CE, Kay J. A systematic series of synthetic chromophoric substrates for aspartic proteinases. The Biochemical journal. 1986;237(3):899–906. 3541904 10.1042/bj2370899 PMC1147073

[54] Dunn BM, Hung S. The two sides of enzyme-substrate specificity: lessons from the aspartic proteinases. Biochimica et biophysica acta. 2000;1477(1–2):231–40. . 10708860 10.1016/s0167-4838(99)00275-7

[55] Reddy GR, Chakrabarti D, Schuster SM, Ferl RJ, Almira EC, Dame JB. Gene sequence tags from Plasmodium falciparum genomic DNA fragments prepared by the "genease" activity of mung bean nuclease. Proceedings of the National Academy of Sciences of the United States of America. 1993;90(21):9867–71. 8234327 10.1073/pnas.90.21.9867 PMC47673

[56] Li T, Yowell CA, Beyer BB, Hung SH, Westling J, Lam MT, et al Recombinant expression and enzymatic subsite characterization of plasmepsin 4 from the four Plasmodium species infecting man. Molecular and biochemical parasitology. 2004;135(1):101–9. . 15287591 10.1016/j.molbiopara.2004.01.010

[57] Beyer BM, Dunn BM. Self-activation of recombinant human lysosomal procathepsin D at a newly engineered cleavage junction, "short" pseudocathepsin D. The Journal of biological chemistry. 1996;271(26):15590–6. . 8663051 10.1074/jbc.271.26.15590

[58] Gasteiger E, Hoogland C, Gattiker A, Duvaud S, Wilkins MR, Appel RD, et al Protein identification and analysis tools on the ExPASy server In: Walker JM, editor. Proteomics Protocols Handbook. Totowa, New Jersey: Humana Press; 2005 p. 571–607.

[59] Dunn BM. The Aspartic Proteinases from the Malaria Parasite: Structure and Function of the Plasmepsins In: Dunn BM, editor. Proteinases as Drug Targets. London: Royal Society of Chemistry; 2011 p. 242–69.

[60] Francis SE, Banerjee R, Goldberg DE. Biosynthesis and maturation of the malaria aspartic hemoglobinases plasmepsins I and II. The Journal of biological chemistry. 1997;272(23):14961–8. . 9169469 10.1074/jbc.272.23.14961

[61] Parr CL, Tanaka T, Xiao H, Yada RY. The catalytic significance of the proposed active site residues in Plasmodium falciparum histoaspartic protease. The FEBS journal. 2008;275(8):1698–707. 10.1111/j.1742-4658.2008.06325.x . 18312598

[62] Kim YM, Lee MH, Piao TG, Lee JW, Kim JH, Lee S, et al Prodomain processing of recombinant plasmepsin II and IV, the aspartic proteases of Plasmodium falciparum, is auto- and trans-catalytic. Journal of biochemistry. 2006;139(2):189–95. 10.1093/jb/mvj018 . 16452306

[63] Tyas L, Gluzman I, Moon RP, Rupp K, Westling J, Ridley RG, et al Naturally-occurring and recombinant forms of the aspartic proteinases plasmepsins I and II from the human malaria parasite Plasmodium falciparum. FEBS letters. 1999;454(3):210–4. . 10431809 10.1016/s0014-5793(99)00805-4

[64] Wyatt DM, Berry C. Activity and inhibition of plasmepsin IV, a new aspartic proteinase from the malaria parasite, Plasmodium falciparum. FEBS letters. 2002;513(2–3):159–62. . 11904142 10.1016/s0014-5793(02)02241-x

[65] Xiao H, Bryksa BC, Bhaumik P, Gustchina A, Kiso Y, Yao SQ, et al The zymogen of plasmepsin V from Plasmodium falciparum is enzymatically active. Molecular and biochemical parasitology. 2014;197(1–2):56–63. 10.1016/j.molbiopara.2014.10.004 . 25447707 PMC6310130

[66] Hsueh WA, Carlson EJ, Israel-Hagman M. Mechanism of acid-activation of renin: role of kallikrein in renin activation. Hypertension. 1981;3(3 Pt 2):I22–9. . 7021413 10.1161/01.hyp.3.3_pt_2.i22

[67] Khan AR, Khazanovich-Bernstein N, Bergmann EM, James MN. Structural aspects of activation pathways of aspartic protease zymogens and viral 3C protease precursors. Proceedings of the National Academy of Sciences of the United States of America. 1999;96(20):10968–75. 10500110 10.1073/pnas.96.20.10968 PMC34228

[68] Shi XP, Chen E, Yin KC, Na S, Garsky VM, Lai MT, et al The pro domain of beta-secretase does not confer strict zymogen-like properties but does assist proper folding of the protease domain. The Journal of biological chemistry. 2001;276(13):10366–73. . 11266439 10.1074/jbc.m009200200

[69] Humphreys MJ, Moon RP, Klinder A, Fowler SD, Rupp K, Bur D, et al The aspartic proteinase from the rodent parasite Plasmodium berghei as a potential model for plasmepsins from the human malaria parasite, Plasmodium falciparum. FEBS letters. 1999;463(1–2):43–8. . 10601635 10.1016/s0014-5793(99)01597-5

[70] Istvan ES, Goldberg DE. Distal substrate interactions enhance plasmepsin activity. The Journal of biological chemistry. 2005;280(8):6890–6. 10.1074/jbc.M412086200 . 15574427

[71] Beyer BB. Targeted Chromogenic Octapeptide Combinatorial Libraries: Exploration of the Primary and Extended Subsite Specificities of Human and Malarial Aspartic Endopeptidases: University of Florida; 2003.

[72] Asojo OA, Gulnik SV, Afonina E, Yu B, Ellman JA, Haque TS, et al Novel uncomplexed and complexed structures of plasmepsin II, an aspartic protease from Plasmodium falciparum. Journal of molecular biology. 2003;327(1):173–81. . 12614616 10.1016/s0022-2836(03)00036-6

[73] Carcache LM, Rodriguez J, Rein KS. The structural basis for kainoid selectivity at AMPA receptors revealed by low-mode docking calculations. Bioorganic & medicinal chemistry. 2003;11(4):551–9. . 12538020 10.1016/s0968-0896(02)00448-0

[74] Bhaumik P, Horimoto Y, Xiao H, Miura T, Hidaka K, Kiso Y, et al Crystal structures of the free and inhibited forms of plasmepsin I (PMI) from Plasmodium falciparum. Journal of structural biology. 2011;175(1):73–84. 10.1016/j.jsb.2011.04.009 21521654 PMC3102120

[75] Bhaumik P, Xiao H, Parr CL, Kiso Y, Gustchina A, Yada RY, et al Crystal structures of the histo-aspartic protease (HAP) from Plasmodium falciparum. Journal of molecular biology. 2009;388(3):520–40. 10.1016/j.jmb.2009.03.011 19285084 PMC2702178

[76] Koshland DE. Application of a Theory of Enzyme Specificity to Protein Synthesis. Proceedings of the National Academy of Sciences of the United States of America. 1958;44(2):98–104. 16590179 10.1073/pnas.44.2.98 PMC335371

[77] Ma B, Kumar S, Tsai CJ, Nussinov R. Folding funnels and binding mechanisms. Protein engineering. 1999;12(9):713–20. 10506280 10.1093/protein/12.9.713

[78] Fischer E. Einfluss der Configuration auf die Wirkung der Enzyme. Ber Dtsch Chem Ges. 1894;27:2985–93.

[79] Moura PA, Dame JB, Fidock DA. Role of Plasmodium falciparum digestive vacuole plasmepsins in the specificity and antimalarial mode of action of cysteine and aspartic protease inhibitors. Antimicrobial agents and chemotherapy. 2009;53(12):4968–78. 10.1128/AAC.00882-09 19752273 PMC2786340

[80] Meyers MJ, Goldberg DE. Recent advances in plasmepsin medicinal chemistry and implications for future antimalarial drug discovery efforts. Current topics in medicinal chemistry. 2012;12(5):445–55. . 22242846 10.2174/156802612799362959 PMC11670882

[81] Ersmark K, Samuelsson B, Hallberg A. Plasmepsins as potential targets for new antimalarial therapy. Medicinal research reviews. 2006;26(5):626–66. 10.1002/med.20082 . 16838300

[82] Dan N, Bhakat S. New paradigm of an old target: an update on structural biology and current progress in drug design towards plasmepsin II. European journal of medicinal chemistry. 2015;95:324–48. 10.1016/j.ejmech.2015.03.049 . 25827401

[83] Noteberg D, Schaal W, Hamelink E, Vrang L, Larhed M. High-speed optimization of inhibitors of the malarial proteases plasmepsin I and II. Journal of combinatorial chemistry. 2003;5(4):456–64. 10.1021/cc0301014 . 12857114

[84] Noteberg D, Hamelink E, Hulten J, Wahlgren M, Vrang L, Samuelsson B, et al Design and synthesis of plasmepsin I and plasmepsin II inhibitors with activity in Plasmodium falciparum-infected cultured human erythrocytes. Journal of medicinal chemistry. 2003;46(5):734–46. 10.1021/jm020951i . 12593654

[85] Nezami A, Luque I, Kimura T, Kiso Y, Freire E. Identification and characterization of allophenylnorstatine-based inhibitors of plasmepsin II, an antimalarial target. Biochemistry. 2002;41(7):2273–80. . 11841219 10.1021/bi0117549

[86] Nezami A, Kimura T, Hidaka K, Kiso A, Liu J, Kiso Y, et al High-affinity inhibition of a family of Plasmodium falciparum proteases by a designed adaptive inhibitor. Biochemistry. 2003;42(28):8459–64. 10.1021/bi034131z . 12859191

[87] Hidaka K, Kimura T, Ruben AJ, Uemura T, Kamiya M, Kiso A, et al Antimalarial activity enhancement in hydroxymethylcarbonyl (HMC) isostere-based dipeptidomimetics targeting malarial aspartic protease plasmepsin. Bioorganic & medicinal chemistry. 2008;16(23):10049–60. 10.1016/j.bmc.2008.10.011 18952439 PMC4447328

[88] Skinner-Adams TS, McCarthy JS, Gardiner DL, Hilton PM, Andrews KT. Antiretrovirals as antimalarial agents. The Journal of infectious diseases. 2004;190(11):1998–2000. 10.1086/425584 . 15529265

[89] Skinner-Adams TS, Andrews KT, Melville L, McCarthy J, Gardiner DL. Synergistic interactions of the antiretroviral protease inhibitors saquinavir and ritonavir with chloroquine and mefloquine against Plasmodium falciparum in vitro. Antimicrobial agents and chemotherapy. 2007;51(2):759–62. 10.1128/AAC.00840-06 17088482 PMC1797772

[90] Hobbs CV, Voza T, Coppi A, Kirmse B, Marsh K, Borkowsky W, et al HIV protease inhibitors inhibit the development of preerythrocytic-stage plasmodium parasites. The Journal of infectious diseases. 2009;199(1):134–41. 10.1086/594369 19032102 PMC3988424

[91] Andrews KT, Fairlie DP, Madala PK, Ray J, Wyatt DM, Hilton PM, et al Potencies of human immunodeficiency virus protease inhibitors in vitro against Plasmodium falciparum and in vivo against murine malaria. Antimicrobial agents and chemotherapy. 2006;50(2):639–48. 10.1128/AAC.50.2.639-648.2006 16436721 PMC1366900

[92] Liu K, Shi H, Xiao H, Chong AG, Bi X, Chang YT, et al Functional profiling, identification, and inhibition of plasmepsins in intraerythrocytic malaria parasites. Angewandte Chemie. 2009;48(44):8293–7. 10.1002/anie.200903747 . 19784986

[93] Bonilla JA, Bonilla TD, Yowell CA, Fujioka H, Dame JB. Critical roles for the digestive vacuole plasmepsins of Plasmodium falciparum in vacuolar function. Molecular microbiology. 2007;65(1):64–75. 10.1111/j.1365-2958.2007.05768.x . 17581121

[94] Liu J, Gluzman IY, Drew ME, Goldberg DE. The role of Plasmodium falciparum food vacuole plasmepsins. The Journal of biological chemistry. 2005;280(2):1432–7. 10.1074/jbc.M409740200 . 15513918

[95] Liu J, Istvan ES, Gluzman IY, Gross J, Goldberg DE. Plasmodium falciparum ensures its amino acid supply with multiple acquisition pathways and redundant proteolytic enzyme systems. Proceedings of the National Academy of Sciences of the United States of America. 2006;103(23):8840–5. 10.1073/pnas.0601876103 16731623 PMC1470969

[96] Sleebs BE, Lopaticki S, Marapana DS, O'Neill MT, Rajasekaran P, Gazdik M, et al Inhibition of Plasmepsin V activity demonstrates its essential role in protein export, PfEMP1 display, and survival of malaria parasites. PLoS biology. 2014;12(7):e1001897 10.1371/journal.pbio.1001897 24983235 PMC4077696

[97] Sleebs BE, Gazdik M, O'Neill MT, Rajasekaran P, Lopaticki S, Lackovic K, et al Transition state mimetics of the Plasmodium export element are potent inhibitors of Plasmepsin V from P. falciparum and P. vivax. Journal of medicinal chemistry. 2014;57(18):7644–62. 10.1021/jm500797g . 25167370

