# Supplementary figures and images for: Enzymatic Characterization of Recombinant Food Vacuole Plasmepsin 4 from the Rodent Malaria Parasite Plasmodium berghei
Source: PLoS One. 2015 Oct 28;10(10):e0141758. doi: 10.1371/journal.pone.0141758 (PMC4624963; doi:10.1371/journal.pone.0141758)

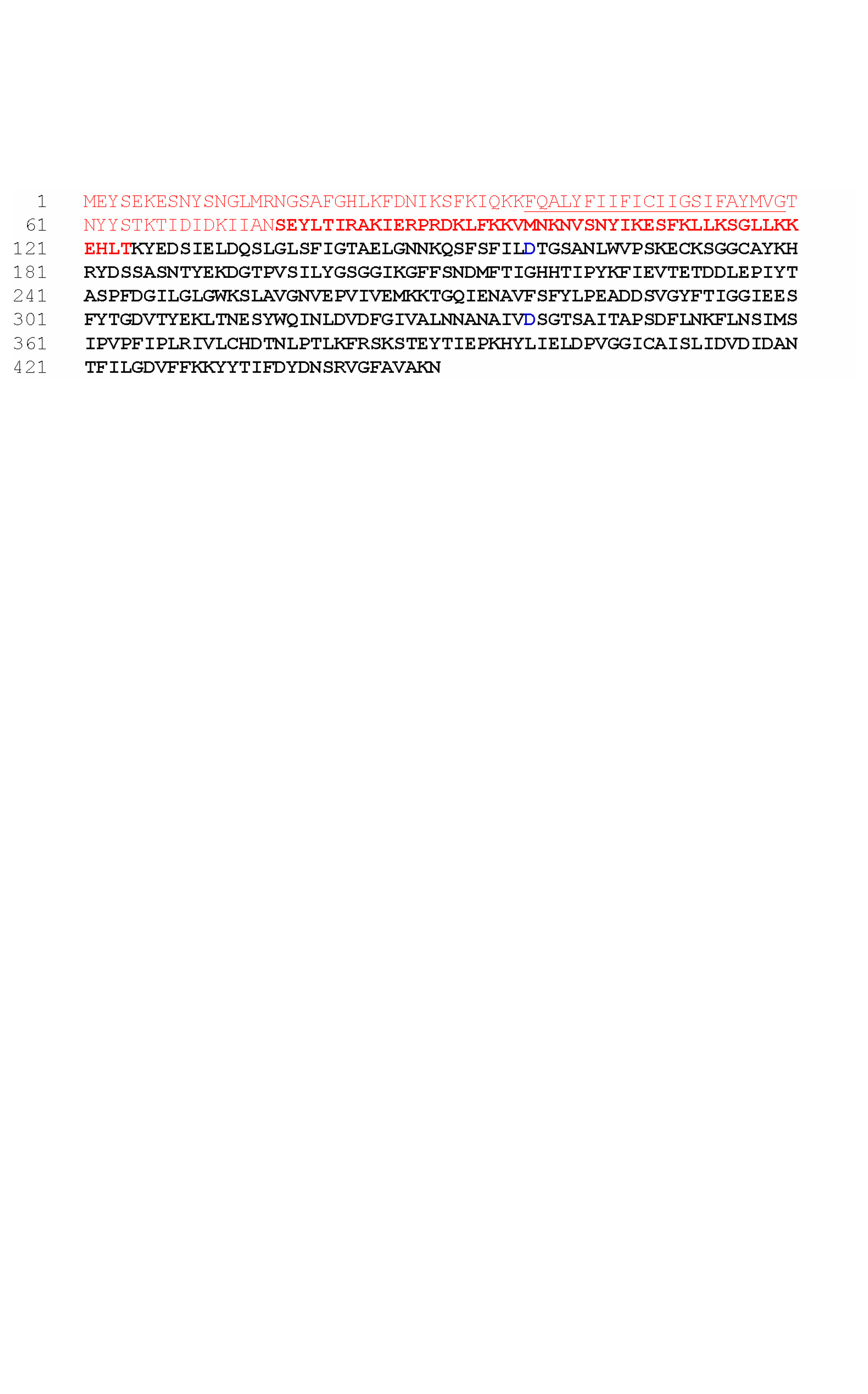

Supplement: S1 Fig — (TIF) [file pone.0141758.s001.tif]

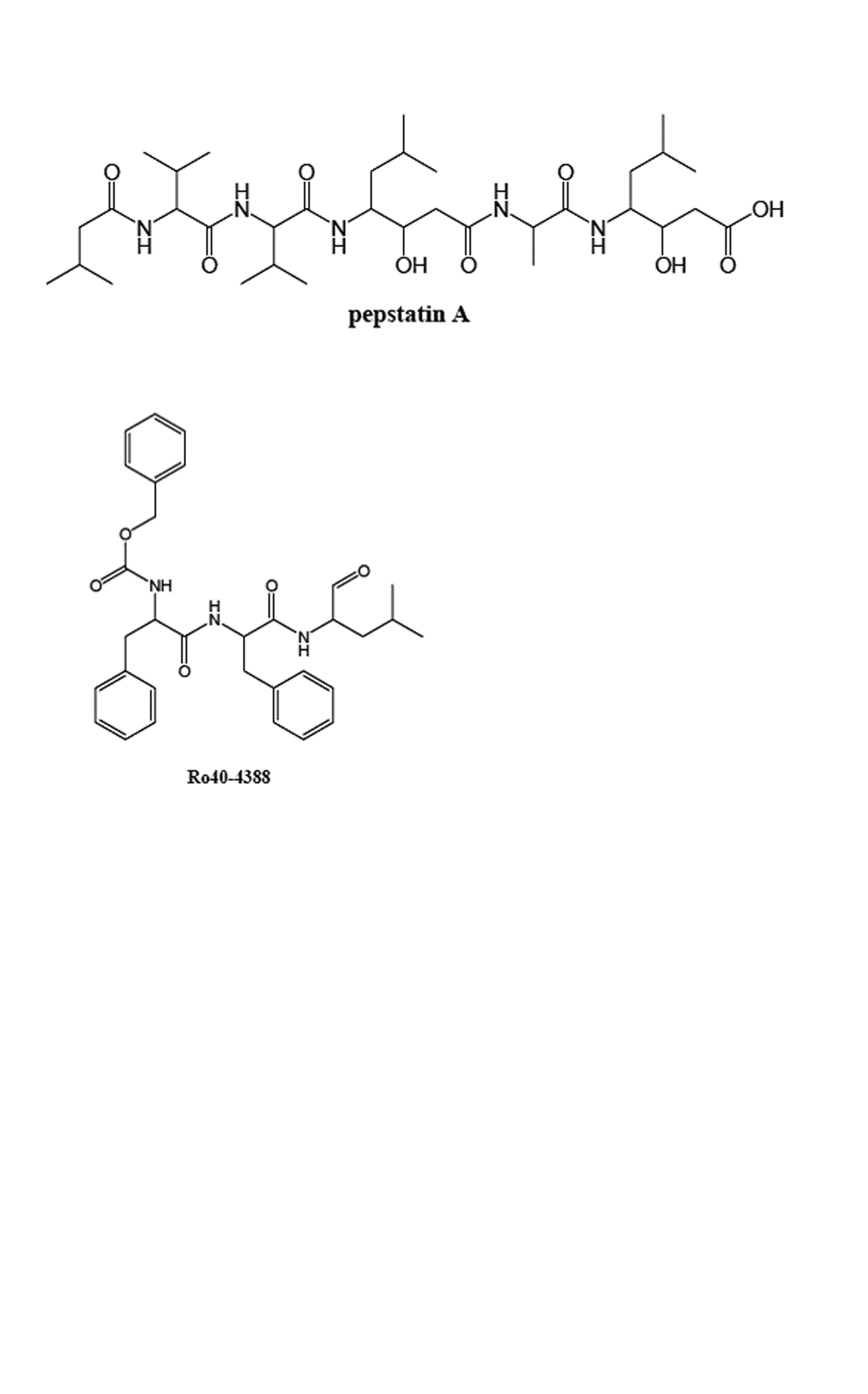

Supplement: S2 Fig — (TIF) [file pone.0141758.s002.tif]
