# Supplementary material for: Enzymatic Characterization of Recombinant Food Vacuole Plasmepsin 4 from the Rodent Malaria Parasite Plasmodium berghei
Source: PLoS One. 2015 Oct 28;10(10):e0141758. doi: 10.1371/journal.pone.0141758 (PMC4624963; doi:10.1371/journal.pone.0141758)
Supplement: S1 File — This file contains the following items: 1) Table A Comparison of primary subsite preferences of PbPM4 and hcatD at S1, 2) Table B Comparison of primary subsite preferences of PbPM4 and hcatD at S1’, 3) Table C Comparison of secondary subsite preferences of PbPM4 and hcatD at S3, 4) Table D Comparison of secondary subsite preferences of PbPM4 and hcatD at S2, 5) Table E Comparison of secondary subsite preferences of PbPM4 and hcatD at S2’, 6) Table F Comparison of secondary subsite preferences of PbPM4 and hcatD at S3’, 7) Table G Comparison of primary subsite preferences of PoPM4 and hcatD at S1, 8) Table H Comparison of primary subsite preferences of PoPM4 and hcatD at S1’, 9) Table I Comparison of secondary subsite preferences of PoPM4 and hcatD at S3, 10) Table J Comparison of secondary subsite preferences of PoPM4 and hcatD at S2, 11) Table K Comparison of secondary subsite preferences of PoPM4 and hcatD at S2’, 12) Table L Comparison of secondary subsite preferences of PoPM4 and hcatD at S3’, 13) Table M Amino acid residues that constitute the S3-S3’ subsite pockets of human and malaria aspartic proteinases, 14) References A. (DOC) [file pone.0141758.s003.doc]

**S1 File. Supporting tables and references.**

Enzymatic characterization of recombinant food vacuole plasmepsin 4 from the rodent malaria parasite *Plasmodium berghei*

Short title: Characterization of plasmepsin 4 of *Plasmodium berghei*

Peng Liu^1,#a,*^, Arthur H. Robbins^1^, Melissa R. Marzahn^1,#b^, Scott H. McClung^2^, Charles A. Yowell^3^, Stanley M. Stevens Jr.^2,#c^, John B. Dame^3^, Ben M. Dunn^1,*^

^1^Department of Biochemistry and Molecular Biology, University of Florida, College of Medicine, Gainesville, Florida, United States of America

^2^Protein Core, Interdisciplinary Center for Biotechnology Research, University of Florida, College of Medicine, Gainesville, Florida, United States of America

^3^Department of Infectious Diseases and Pathology, University of Florida, College of Veterinary Medicine, Gainesville, Florida, United States of America

^#a^Current Address: Department of Neurology and N. Bud Grossman Center for Memory Research and Care, University of Minnesota, Minneapolis, Minnesota, United States of America

^#b^Current Address: Department of Structural Biology, St. Jude Children's Research Hospital, Memphis, Tennessee, United States of America

^#c^Current Address: Department of Cell Biology, Microbiology and Molecular Biology, University of South Florida, Tampa, Florida, United States of America

*****Corresponding authors: [liuxx726@umn.edu](mailto:liuxx726@umn.edu) (PL), [bdunn@ufl.edu](mailto:bdunn@ufl.edu) (BMD)

Table A Comparison of primary subsite preferences of *Pb*PM4 and hcatD at S1

| Amino acid^a^ | hcatD^b^ | *Pb*PM4^b^ | Ratio (*Pb*PM4/hcatD) |
| --- | --- | --- | --- |
| K | 7.128713 | 1.723585 | 0.241781 |
| R | 0.712871 | 0.771029 | 1.081582 |
| H | 2.673267 | 0.13658 | 0.051091 |
| N | 3.366337 | 24.54733 | 7.292001 |
| S | 1.009901 | 4.405226 | 4.362037 |
| Q | 1.346535 | 20.44923 | 15.18656 |
| G | 0.732673 | 1.999542 | 2.729104 |
| A | 9.50495 | 3.873482 | 0.407523 |
| T | 2.376238 | 0.566124 | 0.238244 |
| E | 0.950495 | 1.925281 | 2.025556 |
| D | 3.366337 | 4.07747 | 1.211248 |
| P | 1.841584 | 4.114142 | 2.234023 |
| V | 9.90099 | 5.952326 | 0.601185 |
| Y | 21.78218 | 17.81114 | 0.817693 |
| I | 19.80198 | 6.387807 | 0.322584 |
| L | 75.24752 | 34.38001 | 0.456892 |
| nL | 86.33663 | 41.78318 | 0.483957 |
| **F**^c^ | **100** | **100** | **1** |
| W | 12.67327 | 16.88517 | 1.332346 |

^a^Amino acid substitutes at the P1 position of the P1 combinatorial peptide library.

^b^The initial velocity of hydrolyzing each of the 19 P1 peptide pools by *Pb*PM4 was first determined in three independent experiments. The initial velocities determined in each experiment were then normalized as described in Materials and Methods, and finally, the normalized velocities of the same peptide pool were averaged and reported in this table. The same data processing procedure was applied to human cathepsin D (hcatD). Data for hcatD were obtained from .

^c^The amino acid substitute chosen to comprise compound 1 is in bold font.

**Table B Comparison of primary subsite preferences of *Pb*PM4 and hcatD at S1’**

| Amino acid^a^ | hcatD^b^ | *Pb*PM4^b^ | Ratio (*Pb*PM4/hcatD) |
| --- | --- | --- | --- |
| K | 4.073684 | 2.16 | 0.530233 |
| **R**^c^ | **3.11336** | **11.24** | **3.610247** |
| H | 2.42753 | 3.346667 | 1.378631 |
| N | 36.12955 | 3.786667 | 0.104808 |
| S | 21.76518 | 1.613333 | 0.074124 |
| Q | 1.094737 | 2.12 | 1.936538 |
| G | 12.63158 | 1.946667 | 0.154111 |
| A | 21.1417 | 20 | 0.945998 |
| T | 25.74089 | 20.56 | 0.798729 |
| E | 26.96356 | 2.506667 | 0.092965 |
| D | 11.54656 | 2.653333 | 0.229794 |
| P | 13.52227 | 4.733333 | 0.35004 |
| V | 58.21862 | 73.26667 | 1.258475 |
| Y | 75.89474 | 96.08 | 1.265964 |
| I | 80.38057 | 80.61333 | 1.002896 |
| L | 62.96356 | 89.33333 | 1.41881 |
| nL | 67.76518 | 100 | 1.475684 |
| **F**^c,d^ | **100** | **89.90667** | **0.899067** |
| W | 50.30769 | 14.6667 | 0.29154 |

^a^Amino acid substitutes at the P1’ position of the P1’ combinatorial peptide library.

^b^The initial velocity of hydrolyzing each of the 19 P1’ peptide pools by *Pb*PM4 was first determined in three independent experiments. The initial velocities determined in each experiment were then normalized as described in Materials and Methods, and finally, the normalized velocities of the same peptide pool were averaged and reported in this table. The same data processing procedure was applied to hcatD. Data for hcatD were obtained from .

^c^The amino acid substitute chosen to comprise compound 1 is in bold font.

^d^Although the P1’-phenylalanine peptide pool is not the best recognized one by *Pb*PM4, rather, the P1’-norleucine pool is, data of the secondary subsite preferences of *Pb*PM4 obtained from the P1’-phenylalanine pool were still used to make direct comparisons to the results of hcatD for the design of compound 1.

Table C Comparison of secondary subsite preferences of *Pb*PM4 and hcatD at S3

| Amino acid^a^ | hcatD^b^ | *Pb*PM4^b^ | Ratio (*Pb*PM4/hcatD) |
| --- | --- | --- | --- |
| K | 0.654157 | 0 | 0 |
| R | 0 | 0 | n.a.^d^ |
| H | 0 | 7.509469 | n.a.^d^ |
| N | 6.47891 | 0 | 0 |
| S | 11.30839 | 0 | 0 |
| Q | 14.17774 | 0 | 0 |
| G | 6.525338 | 0 | 0 |
| A | 10.45289 | 0 | 0 |
| T | 9.523996 | 0 | 0 |
| E | 15.17356 | 14.56542 | 0.959921 |
| D | 10.93647 | 0 | 0 |
| P | 9.264258 | 4.668455 | 0.503921 |
| V | 36.99321 | 20.10888 | 0.543583 |
| **Y**^c^ | **64.77044** | **93.86472** | **1.449191** |
| I | 71.67673 | 97.32425 | 1.357822 |
| L | 75.57094 | 97.6466 | 1.292118 |
| nL | 78.43992 | 60.0903 | 0.766068 |
| F | 100 | 100 | 1 |
| W | 83.58452 | 73.8111 | 0.883071 |

^a^Amino acid substitutes at the P3 position of the P1 combinatorial peptide library.

^b^The abundances (in arbitrary units) of penta-peptides obtained from hydrolyzing the P1-phenylalanine peptide pool by *Pb*PM4 were first determined in at least three independent experiments. The abundances of penta-peptides determined in each experiment were then normalized as described in Materials and Methods, and finally, the normalized relative abundances of the same penta-peptide were averaged and reported in this table. The same data processing procedure was applied to hcatD. Data for hcatD were obtained from .

^c^The amino acid substitute chosen to comprise compound 1 is in bold font.

^d^n.a. = not applicable.

Table D Comparison of secondary subsite preferences of *Pb*PM4 and hcatD at S2

| Amino acid^a^ | hcatD^b^ | *Pb*PM4^b^ | Ratio (*Pb*PM4/hcatD) |
| --- | --- | --- | --- |
| K | 1.753513 | 1.670412 | 0.952609 |
| R | 2.806395 | 0.997009 | 0.355263 |
| H | 0.211634 | 1.222169 | 5.774921 |
| N | 6.199317 | 22.36853 | 3.608225 |
| S | 33.52579 | 56.93993 | 1.698392 |
| Q | 9.858637 | 22.08492 | 2.240159 |
| G | 2.489931 | 19.85232 | 7.973042 |
| A | 26.17768 | 28.57964 | 1.091756 |
| T | 28.0553 | 18.27051 | 0.651232 |
| **E**^c^ | **43.95856** | **100** | **2.27487** |
| D | 14.47841 | 28.70081 | 1.982318 |
| P | 0 | 21.82884 | n.a.^d^ |
| V | 32.38007 | 45.8864 | 1.417119 |
| Y | 70.79179 | 43.50109 | 0.614493 |
| I | 86.51342 | 90.5668 | 1.046853 |
| L | 68.26143 | 41.09583 | 0.602036 |
| nL | 89.95753 | 39.94707 | 0.444066 |
| F | 100 | 51.94103 | 0.51941 |
| W | 60.19735 | 31.06716 | 0.516089 |

^a^Amino acid substitutes at the P2 position of the P1’ combinatorial peptide library.

^b^The abundances (in arbitrary units) of penta-peptides obtained from hydrolyzing the P1’-phenylalanine peptide pool by *Pb*PM4 were first determined in at least three independent experiments. The abundances of penta-peptides determined in each experiment were then normalized as described in Materials and Methods, and finally, the normalized relative abundances of the same penta-peptide were averaged and reported in this table. The same data processing procedure was applied to hcatD. Data for hcatD were obtained from .

^c^The amino acid substitute chosen to comprise compound 1 is in bold font.

^d^n.a. = not applicable.

Table E Comparison of secondary subsite preferences of *Pb*PM4 and hcatD at S2’

| Amino acid^a^ | hcatD^b^ | *Pb*PM4^b^ | Ratio (*Pb*PM4/hcatD) |
| --- | --- | --- | --- |
| K | 17.08068 | 4.94799 | 0.289683 |
| R | 32.60579 | 7.800998 | 0.239252 |
| H | 2.695764 | 6.609637 | 2.451861 |
| N | 3.160365 | 56.08831 | 17.74742 |
| S | 0 | 100 | n.a.^d^ |
| **Q**^c^ | **7.683888** | **98.8491** | **12.86446** |
| G | 33.76315 | 58.85395 | 1.743142 |
| A | 40.21338 | 42.54136 | 1.057891 |
| T | 36.11476 | 83.94136 | 2.324295 |
| E | 47.53144 | 83.76173 | 1.762238 |
| D | 38.84493 | 18.26976 | 0.470325 |
| P | 94.4771 | 7.598661 | 0.080429 |
| V | 5.658894 | 62.71437 | 11.08244 |
| Y | 54.87631 | 88.47288 | 1.612224 |
| I | 100 | 56.7233 | 0.567233 |
| L | 78.27911 | 78.13513 | 0.998161 |
| nL | 53.88823 | 56.72046 | 1.052557 |
| F | 57.65356 | 70.958 | 1.230765 |
| W | 66.39613 | 42.30112 | 0.637102 |

^a^Amino acid substitutes at the P2’ position of the P1 combinatorial peptide library.

^b^The abundances (in arbitrary units) of tri-peptides obtained from hydrolyzing the P1-phenylalanine peptide pool by *Pb*PM4 were first determined in at least three independent experiments. The abundances of tri-peptides determined in each experiment were then normalized as described in Materials and Methods, and finally, the normalized relative abundances of the same tri-peptide were averaged and reported in this table. The same data processing procedure was applied to hcatD. Data for hcatD were obtained from .

^c^The amino acid substitute chosen to comprise compound 1 is in bold font.

^d^n.a. = not applicable.

Table F Comparison of secondary subsite preferences of *Pb*PM4 and hcatD at S3’

| Amino acid^a^ | hcatD^b^ | *Pb*PM4^b^ | Ratio (*Pb*PM4/hcatD) |
| --- | --- | --- | --- |
| K | 1.67779 | 0.064306 | 0.038328 |
| R | 3.625303 | 0.221725 | 0.06116 |
| H | 1.890749 | 0.433568 | 0.22931 |
| N | 2.339007 | 0.285242 | 0.12195 |
| S | 3.312609 | 0.55716 | 0.168194 |
| Q | 8.222782 | 0.46636 | 0.056716 |
| G | 6.657095 | 0.054393 | 0.008171 |
| A | 15.03259 | 0.165076 | 0.010981 |
| T | 8.293029 | 4.193214 | 0.505631 |
| E | 22.99352 | 4.169324 | 0.181326 |
| D | 50.99467 | 9.323249 | 0.182828 |
| P | 19.7682 | 1.62867 | 0.082388 |
| V | 66.01569 | 0.732816 | 0.011101 |
| Y | 13.50122 | 3.267351 | 0.242004 |
| I | 100 | 100 | 1 |
| L | 66.84166 | 77.72072 | 1.162759 |
| nL | 52.10569 | 71.83434 | 1.378627 |
| **F**^c^ | **49.98832** | **98.65144** | **1.97349** |
| W | 39.16801 | 73.60494 | 1.879211 |

^a^Amino acid substitutes at the P3’ position of the P1’ combinatorial peptide library.

^b^The abundances (in arbitrary units) of tri-peptides obtained from hydrolyzing the P1’-phenylalanine peptide pool by *Pb*PM4 were first determined in at least three independent experiments. The abundances of tri-peptides determined in each experiment were then normalized as described in Materials and Methods, and finally, the normalized relative abundances of the same tri-peptide were averaged and reported in this table. The same data processing procedure was applied to hcatD. Data for hcatD were obtained from .

^c^The amino acid substitute chosen to comprise compound 1 is in bold font.

Table G Comparison of primary subsite preferences of *Po*PM4 and hcatD at S1

| Amino acid^a^ | hcatD^b^ | *Po*PM4^b^ | Ratio (*Po*PM4/hcatD) |
| --- | --- | --- | --- |
| K | 7.128713 | 0.768595 | 0.107817 |
| R | 0.712871 | 0.495868 | 0.695592 |
| H | 2.673267 | 2.421488 | 0.905816 |
| N | 3.366337 | 0.570248 | 0.169397 |
| S | 1.009901 | 2.867769 | 2.839653 |
| Q | 1.346535 | 0.099174 | 0.073651 |
| G | 0.732673 | 2.603306 | 3.553161 |
| A | 9.50495 | 1.190083 | 0.125207 |
| T | 2.376238 | 2.157025 | 0.907748 |
| E | 0.950495 | 0.107438 | 0.113034 |
| D | 3.366337 | 0.826446 | 0.245503 |
| P | 1.841584 | 0.92562 | 0.502622 |
| V | 9.90099 | 2.454545 | 0.247909 |
| Y | 21.78218 | 14.95868 | 0.686739 |
| I | 19.80198 | 0.115702 | 0.005843 |
| L | 75.24752 | 27.68595 | 0.367932 |
| nL | 86.33663 | 53.96694 | 0.625076 |
| **F**^c^ | **100** | **100** | **1** |
| W | 12.67327 | 3.115702 | 0.245848 |

^a^Amino acid substitutes at the P1 position of the P1 combinatorial peptide library.

^b^The initial velocity of hydrolyzing each of the 19 P1 peptide pools by *Po*PM4 was first determined in three independent experiments. The initial velocities determined in each experiment were then normalized as described in Materials and Methods, and finally, the normalized velocities of the same peptide pool were averaged and reported in this table. The same data processing procedure was applied to hcatD. Data for *Po*PM4 and hcatD were obtained from .

^c^The amino acid substitute chosen to comprise compound 6 is in bold font.

Table H Comparison of primary subsite preferences of *Po*PM4 and hcatD at S1’

| Amino acid^a^ | hcatD^b^ | *Po*PM4^b^ | Ratio (*Po*PM4/hcatD) |
| --- | --- | --- | --- |
| K | 4.073684 | 18.17378 | 4.461265 |
| R | 3.11336 | 27.24595 | 8.751301 |
| H | 2.42753 | 13.3137 | 5.484462 |
| N | 36.12955 | 17.96024 | 0.497107 |
| S | 21.76518 | 16.10457 | 0.739923 |
| Q | 1.094737 | 19.21944 | 17.55622 |
| G | 12.63158 | 1.580265 | 0.125104 |
| A | 21.1417 | 26.98822 | 1.27654 |
| T | 25.74089 | 28.07806 | 1.090796 |
| E | 26.96356 | 27.47423 | 1.018939 |
| D | 11.54656 | 7.309278 | 0.633026 |
| P | 13.52227 | 3.900589 | 0.288457 |
| V | 58.21862 | 26.02356 | 0.446997 |
| **Y**^c^ | **75.89474** | **100** | **1.317614** |
| I | 80.38057 | 27.04713 | 0.336488 |
| L | 62.96356 | 31.34757 | 0.497868 |
| nL | 67.76518 | 38.07806 | 0.561912 |
| F^d^ | 100 | 48.97644 | 0.489764 |
| W | 50.30769 | 11.86303 | 0.23581 |

^a^Amino acid substitutes at the P1’ position of the P1’ combinatorial peptide library.

^b^The initial velocity of hydrolyzing each of the 19 P1’ peptide pools by *Po*PM4 was first determined in three independent experiments. The initial velocities determined in each experiment were then normalized as described in Materials and Methods, and finally, the normalized velocities of the same peptide pool were averaged and reported in this table. The same data processing procedure was applied to hcatD. Data for *Po*PM4 and hcatD were obtained from .

^c^The amino acid substitute chosen to comprise compound 6 is in bold font.

^d^Although the P1’-phenylalanine peptide pool is not the best recognized one by *Po*PM4, rather, the P1’-tyrosine pool is, data of the secondary subsite preferences of *Po*PM4 obtained from the P1’-phenylalanine pool were still used to make direct comparisons to the results of hcatD for the design of compound 6.

Table I Comparison of secondary subsite preferences of *Po*PM4 and hcatD at S3

| Amino acid^a^ | hcatD^b^ | *Po*PM4^b^ | Ratio (*Po*PM4/hcatD) |
| --- | --- | --- | --- |
| K | 0.654157 | 0 | 0 |
| R | 0 | 0 | n.a.^d^ |
| H | 0 | 0 | n.a.^d^ |
| N | 6.47891 | 1.253381 | 0.193456 |
| S | 11.30839 | 2.05028 | 0.181306 |
| Q | 14.17774 | 0 | 0 |
| G | 6.525338 | 0 | 0 |
| A | 10.45289 | 6.957783 | 0.665632 |
| T | 9.523996 | 5.849368 | 0.614172 |
| E | 15.17356 | 0 | 0 |
| D | 10.93647 | 5.090211 | 0.465435 |
| P | 9.264258 | 6.801467 | 0.734162 |
| V | 36.99321 | 31.17581 | 0.842744 |
| Y | 64.77044 | 26.44416 | 0.408275 |
| I | 71.67673 | 71.50849 | 0.997653 |
| **L^c^** | **75.57094** | **86.92314** | **1.150219** |
| nL | 78.43992 | 87.37313 | 1.113886 |
| F | 100 | 100 | 1 |
| W | 83.58452 | 37.42042 | 0.447696 |

^a^Amino acid substitutes at the P3 position of the P1 combinatorial peptide library.

^b^The abundances (in arbitrary units) of penta-peptides obtained from hydrolyzing the P1-phenylalanine peptide pool by *Po*PM4 were first determined in at least three independent experiments. The abundances of penta-peptides determined in each experiment were then normalized as described in Materials and Methods, and finally, the normalized relative abundances of the same penta-peptide were averaged and reported in this table. The same data processing procedure was applied to hcatD. Data for *Po*PM4 and hcatD were obtained from .

^c^The amino acid substitute chosen to comprise compound 6 is in bold font.

^d^n.a. = not applicable.

Table J Comparison of secondary subsite preferences of *Po*PM4 and hcatD at S2

| Amino acid^a^ | hcatD^b^ | *Po*PM4^b^ | Ratio (*Po*PM4/hcatD) |
| --- | --- | --- | --- |
| K | 1.753513 | 10.95181 | 6.245637 |
| R | 2.806395 | 4.347273 | 1.549059 |
| H | 0.211634 | 9.352479 | 44.19178 |
| N | 6.199317 | 19.14287 | 3.0879 |
| S | 33.52579 | 83.73069 | 2.497501 |
| Q | 9.858637 | 42.59146 | 4.320218 |
| G | 2.489931 | 37.87436 | 15.21101 |
| A | 26.17768 | 39.1508 | 1.495579 |
| T | 28.0553 | 30.15357 | 1.07479 |
| **E**^c^ | **43.95856** | **100** | **2.27487** |
| D | 14.47841 | 15.6011 | 1.077542 |
| P | 0 | 1.727959 | n.a.^d^ |
| V | 32.38007 | 30.52866 | 0.942823 |
| Y | 70.79179 | 23.49554 | 0.331896 |
| I | 86.51342 | 67.77013 | 0.783348 |
| L | 68.26143 | 43.60947 | 0.63886 |
| nL | 89.95753 | 56.12296 | 0.623883 |
| F | 100 | 47.6705 | 0.476705 |
| W | 60.19735 | 34.76698 | 0.57755 |

^a^Amino acid substitutes at the P2 position of the P1’ combinatorial peptide library.

^b^The abundances (in arbitrary units) of penta-peptides obtained from hydrolyzing the P1’-phenylalanine peptide pool by *Po*PM4 were first determined in at least three independent experiments. The abundances of penta-peptides determined in each experiment were then normalized as described in Materials and Methods, and finally, the normalized relative abundances of the same penta-peptide were averaged and reported in this table. The same data processing procedure was applied to hcatD. Data for *Po*PM4 and hcatD were obtained from .

^c^The amino acid substitute chosen to comprise compound 6 is in bold font.

^d^n.a. = not applicable.

Table K Comparison of secondary subsite preferences of *Po*PM4 and hcatD at S2’

| Amino acid^a^ | hcatD^b^ | *Po*PM4^b^ | Ratio (*Po*PM4/hcatD) |
| --- | --- | --- | --- |
| K | 17.08068 | 45.9088 | 2.687762 |
| **R**^c^ | **32.60579** | **87.68963** | **2.689388** |
| H | 2.695764 | 22.72927 | 8.431476 |
| N | 3.160365 | 18.66968 | 5.907444 |
| S | 0 | 0 | n.a.^d^ |
| Q | 7.683888 | 14.40821 | 1.87512 |
| G | 33.76315 | 50.53613 | 1.496784 |
| A | 40.21338 | 92.23427 | 2.293621 |
| T | 36.11476 | 52.62281 | 1.4571 |
| E | 47.53144 | 57.28438 | 1.205189 |
| D | 38.84493 | 36.93092 | 0.950727 |
| P | 94.4771 | 65.92238 | 0.69776 |
| V | 5.658894 | 32.40232 | 5.72591 |
| Y | 54.87631 | 51.77135 | 0.943419 |
| I | 100 | 88.76312 | 0.887631 |
| L | 78.27911 | 79.64287 | 1.017422 |
| nL | 53.88823 | 84.20453 | 1.562577 |
| F | 57.65356 | 100 | 1.734499 |
| W | 66.39613 | 50.54033 | 0.761194 |

^a^Amino acid substitutes at the P2’ position of the P1 combinatorial peptide library.

^b^The abundances (in arbitrary units) of tri-peptides obtained from hydrolyzing the P1-phenylalanine peptide pool by *Po*PM4 were first determined in at least three independent experiments. The abundances of tri-peptides determined in each experiment were then normalized as described in Materials and Methods, and finally, the normalized relative abundances of the same tri-peptide were averaged and reported in this table. The same data processing procedure was applied to hcatD. Data for *Po*PM4 and hcatD were obtained from .

^c^The amino acid substitute chosen to comprise compound 6 is in bold font.

^d^n.a. = not applicable.

Table L Comparison of secondary subsite preferences of *Po*PM4 and hcatD at S3’

| Amino acid^a^ | hcatD^b^ | *Po*PM4^b^ | Ratio (*Po*PM4/hcatD) |
| --- | --- | --- | --- |
| K | 1.67779 | 5.610079 | 3.343731 |
| R | 3.625303 | 7.253446 | 2.000783 |
| H | 1.890749 | 7.807881 | 4.129517 |
| N | 2.339007 | 7.320502 | 3.129748 |
| S | 3.312609 | 22.61145 | 6.825875 |
| Q | 8.222782 | 13.71606 | 1.668055 |
| G | 6.657095 | 15.88167 | 2.385676 |
| A | 15.03259 | 26.75383 | 1.779723 |
| T | 8.293029 | 15.9144 | 1.919009 |
| E | 22.99352 | 39.89485 | 1.735047 |
| D | 50.99467 | 56.60086 | 1.109937 |
| P | 19.7682 | 39.00061 | 1.972896 |
| **V**^c^ | **66.01569** | **70.49101** | **1.067792** |
| Y | 13.50122 | 19.77429 | 1.46463 |
| I | 100 | 100 | 1 |
| L | 66.84166 | 65.75851 | 0.983795 |
| nL | 52.10569 | 54.24627 | 1.041081 |
| F | 49.98832 | 54.56209 | 1.091497 |
| W | 39.16801 | 54.24771 | 1.385001 |

^a^Amino acid substitutes at the P3’ position of the P1’ combinatorial peptide library.

^b^The abundances (in arbitrary units) of tri-peptides obtained from hydrolyzing the P1’-phenylalanine peptide pool by *Po*PM4 were first determined in at least three independent experiments. The abundances of tri-peptides determined in each experiment were then normalized as described in Materials and Methods, and finally, the normalized relative abundances of the same tri-peptide were averaged and reported in this table. The same data processing procedure was applied to hcatD. Data for *Po*PM4 and hcatD were obtained from .

^c^The amino acid substitute chosen to comprise compound 6 is in bold font.

Table M Amino acid residues that constitute the S3-S3’ subsite pockets of human and malaria aspartic proteinases

| S3 | hPepA | hCatD | hCatE | *Pf*PM1 | *Pf*PM2 | *Pf*PM4 | *Pv*PM4 | *Po*PM4 | *Pm*PM4 | *Pb*PM4 |
| --- | --- | --- | --- | --- | --- | --- | --- | --- | --- | --- |
| 9 | Y | Y | Y | V | F | V | V | I | V | S |
| 12 | M | A | M | V | I | L | I | L | L | L |
| 13 | E | Q | E | M | M | M | M | V | M | S |
| 111 | F | T | T | A | T | I | I | V | L | I |
| 115 | A | A | A | G | S | S | V | I | A | S |
| 117 | F | F | F | F | F | F | F | F | F | F |
| S2 | hPepA | hCatD | hCatE | *Pf*PM1 | *Pf*PM2 | *Pf*PM4 | *Pv*PM4 | *Po*PM4 | *Pm*PM4 | *Pb*PM4 |
| 76 | G | G | G | V | V | G | G | G | G | G |
| 219 | S | S | S | S | S | S | T | S | S | S |
| 222 | T | V | T | T | T | T | T | T | T | T |
| 287 | Q | M | Q | I | I | L | L | I | L | I |
| 289 | M | M | L | V | L | V | V | V | V | V |
| S1 | hPepA | hCatD | hCatE | *Pf*PM1 | *Pf*PM2 | *Pf*PM4 | *Pv*PM4 | *Po*PM4 | *Pm*PM4 | *Pb*PM4 |
| 30 | V | V | I | I | I | I | I | L | I | I |
| 32 | D | D | D | D | D | D | D | D | D | D |
| 34 | G | G | G | G | G | G | G | G | G | G |
| 75 | Y | Y | Y | Y | Y | Y | Y | Y | Y | Y |
| 76 | G | G | G | V | G | G | G | G | G | G |
| 77 | T | S | T | S | S | S | S | S | S | S |
| S1’ | hPepA | hCatD | hCatE | *Pf*PM1 | *Pf*PM2 | *Pf*PM4 | *Pv*PM4 | *Po*PM4 | *Pm*PM4 | *Pb*PM4 |
| 189 | Y | Y | Y | Y | Y | Y | Y | Y | F | Y |
| 213 | I | I | I | I | I | V | I | I | I | I |
| 215 | D | D | D | D | D | D | D | D | D | D |
| 217 | G | G | G | G | G | G | G | G | G | G |
| 218 | T | T | T | T | T | T | T | T | T | T |
| 300 | I | I | I | I | I | I | I | I | I | I |
| S2’ | hPepA | hCatD | hCatE | *Pf*PM1 | *Pf*PM2 | *Pf*PM4 | *Pv*PM4 | *Po*PM4 | *Pm*PM4 | *Pb*PM4 |
| 73 | I | I | I | M | M | I | I | L | I | I |
| 74 | T | H | Q | N | N | S | T | L | T | L |
| 128 | I | I | L | L | L | L | L | L | L | L |
| 130 | S | V | V | I | I | I | I | I | I | V |
| S3’ | hPepA | hCatD | hCatE | *Pf*PM1 | *Pf*PM2 | *Pf*PM4 | *Pv*PM4 | *Po*PM4 | *Pm*PM4 | *Pb*PM4 |
| 76 | G | G | G | V | V | G | G | G | G | G |
| 289 | M | M | L | V | L | V | V | V | V | V |
| 291 | L | I | I | L | F | I | I | I | I | I |
| 292 | P | P | H | N | P | D | D | D | D | D |

hPepA, human pepsin A; hcatD, human cathepsin D; hcatE, human cathepsin E; *Pf*PM1, *Pf*PM2 and *Pf*PM4, plasmepsins 1, 2 and 4 from *Plasmodium falciparum*, respectively; *Pv*PM4, plasmepsin 4 from *P. vivax*; *Po*PM4, plasmepsin 4 from *P. ovalae*; and *Pm*PM4, plasmepsin 4 from *P. malariae*. Amino Acid residues are numbered based on the sequence of human pepsin A.

Amino acid residues that constitute subsites of enzymes were identified from crystal structures of hPepA , hcatD , *Pf*PM1 , *Pf*PM2 , *Pf*PM4 , *Pv*PM4 and *Pm*PM4 . Residues of hcatE, *Po*PM4 and *Pb*PM4 listed in this table were identified by sequence alignment with enzymes of known structures.

References A

1. Beyer BB. Targeted Chromogenic Octapeptide Combinatorial Libraries: Exploration of the Primary and Extended Subsite Specificities of Human and Malarial Aspartic Endopeptidases: University of Florida; 2003.

2. Fujinaga M, Chernaia MM, Tarasova NI, Mosimann SC, James MN. Crystal structure of human pepsin and its complex with pepstatin. Protein science : a publication of the Protein Society. 1995;4(5):960-72. doi: 10.1002/pro.5560040516. PubMed PMID: 7663352; PubMed Central PMCID: PMC2143119.

3. Baldwin ET, Bhat TN, Gulnik S, Hosur MV, Sowder RC, 2nd, Cachau RE, et al. Crystal structures of native and inhibited forms of human cathepsin D: implications for lysosomal targeting and drug design. Proceedings of the National Academy of Sciences of the United States of America. 1993;90(14):6796-800. PubMed PMID: 8393577; PubMed Central PMCID: PMC47019.

4. Bhaumik P, Horimoto Y, Xiao H, Miura T, Hidaka K, Kiso Y, et al. Crystal structures of the free and inhibited forms of plasmepsin I (PMI) from Plasmodium falciparum. Journal of structural biology. 2011;175(1):73-84. doi: 10.1016/j.jsb.2011.04.009. PubMed PMID: 21521654; PubMed Central PMCID: PMC3102120.

5. Asojo OA, Gulnik SV, Afonina E, Yu B, Ellman JA, Haque TS, et al. Novel uncomplexed and complexed structures of plasmepsin II, an aspartic protease from Plasmodium falciparum. Journal of molecular biology. 2003;327(1):173-81. PubMed PMID: 12614616.

6. Bernstein NK, Cherney MM, Yowell CA, Dame JB, James MN. Structural insights into the activation of P. vivax plasmepsin. Journal of molecular biology. 2003;329(3):505-24. PubMed PMID: 12767832.

7. Clemente JC, Govindasamy L, Madabushi A, Fisher SZ, Moose RE, Yowell CA, et al. Structure of the aspartic protease plasmepsin 4 from the malarial parasite Plasmodium malariae bound to an allophenylnorstatine-based inhibitor. Acta crystallographica Section D, Biological crystallography. 2006;62(Pt 3):246-52. doi: 10.1107/S0907444905041260. PubMed PMID: 16510971.

**S1 Fig. Primary structure of pro*Pb*PM4.** The 124-amino acid-long prosegment sequence is highlighted in red. The putative transmembrane motif is underlined. The two aspartates directing catalysis are bolded and highlighted in blue. The sequence of the semi-pro*Pb*PM4 construct is bolded.

**S2 Fig. Structures of inhibitors tested in Table 3.**
